# Transcranial random noise stimulation (tRNS) over the left dorsolateral prefrontal cortex ameliorates emotion dysregulation and executive function: a single-blind, randomized, sham-controlled clinical trial

**DOI:** 10.1186/s40359-026-05214-w

**Published:** 2026-07-17

**Authors:** Mahdi Hajiaqaei, Alireza Mohammadi

**Affiliations:** 1 Student Research Committee, Baqiyatallah University of Medical Sciences, Tehran, Iran; 2 Neuroscience Research Center, Baqiyatallah University of Medical Sciences, Tehran, Iran

**Keywords:** Transcranial random noise stimulation, Dorsolateral prefrontal cortex, Emotion dysregulation, Executive function

## Abstract

**Background:**

Emotion regulation involves executive functions like inhibitory control and working memory, strongly linked to the left dorsolateral prefrontal cortex (DLPFC). This study examined whether transcranial random noise stimulation (tRNS) over the DLPFC could mitigate negative affect and associated regulatory failures induced by exposure to traumatic images in healthy individuals, serving as an experimental model of acute emotional challenge that taxes emotion regulation systems.

**Methods:**

Sixty participants were randomized to six groups (*n* = 10): Sham, Emotion Dysregulation (Edys), Left tRNS (L-tRNS), Bilateral tRNS (LR-tRNS), Edys + L-tRNS, and Edys + LR-tRNS. Edys was induced via 50 traumatic images. tRNS (2 mA, 20 min) was applied unilaterally (Left DLPFC) or bilaterally (with anodal electrodes over F3/F4 and cathodal placements over F1/F5/AF3/FC3 or F2/F6/AF4/FC4, respectively). Assessments pre- and post-intervention included the Difficulties in Emotion Regulation Scale (DERS), Paced Auditory Serial Addition Test (PASAT), Emotion Regulation Questionnaire (ERQ), and Cognitive Emotion Regulation Questionnaire (CERQ).

**Results:**

Emotion dysregulation induction significantly increased DERS scores and impaired PASAT performance. Active left unilateral tRNS following induction produced the greatest improvements in emotion regulation and executive function measures compared to sham and induction-only groups. Bilateral tRNS effects were smaller and inconsistent. Unilateral Left DLPFC stimulation was more effective than bilateral in transiently improving these processes.

**Conclusions:**

tRNS over the Left DLPFC may facilitate regulatory and executive functions during transient emotion dysregulation in healthy individuals. These preliminary findings from a non-clinical sample suggest that neuromodulation may facilitate emotion regulation during emotional challenge. The small sample size necessitates caution and replication.

**Trial registration:**

Iranian Registry of Clinical Trials, IRCT20170417033485N2, registered on 2025/07/25.

**Supplementary Information:**

The online version contains supplementary material available at https://doi.org/10.1186/s40359-026-05214-w.

## Background

Emotion regulation is essential for achieving personal and social goals, particularly when individuals face stressors that require adaptive responses [1, 2]. Emotion regulation has been broadly defined as the monitoring, evaluation, and modulation of emotional reactions to achieve goals [3]. Cognitively, high-level executive and social processes are required for emotion regulation, including inhibitory control, abstract thought, decision-making, working memory (WM), and perspective taking [4].

Evidence indicates that structural brain development in regions responsible for emotion regulation continues into adulthood. For example, the prefrontal cortex (PFC) plays a central role in the generation and maintenance of emotion regulation [3]. The regions most consistently implicated in cognitive control processes include the orbitofrontal cortex (OFC), dorsomedial prefrontal cortex, anterior cingulate gyrus, dorsolateral prefrontal cortex (DLPFC), and ventrolateral prefrontal cortex (vlPFC) [5]. The DLPFC is connected to subcortical regions, including the thalamus, dorsal striatum (caudate nucleus), hippocampus, and primary and secondary cortical association areas such as the posterior temporal, parietal, and occipital cortices [5]. Limbic regions, particularly the amygdala and hippocampus, interact closely with the DLPFC in emotion regulation. Prefrontal-limbic connectivity strength predicts individual differences in reappraisal success, and amygdala hyperactivity is associated with impaired regulatory capacity [6]. Moreover, recent studies have shown that the hippocampus plays a crucial role in emotional and motivational processes during adolescence [3]. Disruptions or abnormal connectivity in these regions may contribute to emotion dysregulation, impulsivity, and aggressive behavior [7]. Adaptive regulatory strategies, such as cognitive reappraisal (CR), are associated with enhanced executive functioning and positive outcomes, including well-being and social functioning [8, 9], whereas maladaptive strategies, such as expressive suppression (ES), are linked to poorer functioning [10, 11].

Indeed, a variety of categories of psychopathology are characterized by low use of CR and high use of ES, such as depression and social anxiety disorder [12, 13]. Thus, reduced CR and increased ES may be indicative of emotion dysregulation. Emotion dysregulation occurs in many psychiatric conditions [14] and it seems that employing a therapeutic strategy aimed at improving executive function impairment and emotion dysregulation could be beneficial in this pathological condition.

In the present study, we induced a transient state of heightened negative affect and emotional distress in healthy adults by exposing them to 50 traumatic images. Throughout this manuscript, we operationalize induced emotion dysregulation as a significant pre-to-post increase in DERS scores following this induction, reflecting a state-level regulatory challenge rather than a clinical or trait-level deficit. This procedure models the acute emotional demands that can temporarily overwhelm regulatory capacity, sharing certain features—such as heightened emotional reactivity and increased reliance on maladaptive strategies—with the affective instability observed in clinical conditions [15].

To investigate potential neuromodulatory interventions, we applied transcranial random noise stimulation (tRNS) over the DLPFC. The left DLPFC stimulation has been associated with enhanced cognitive control and emotion regulation processes, whereas bilateral stimulation may introduce competing or less consistent effects [16–18]. tRNS is a non-invasive brain stimulation technique that applies random electrical oscillations to the scalp. Unlike transcranial direct current stimulation (tDCS), which delivers a constant current, tRNS is thought to enhance cortical excitability by increasing the signal-to-noise ratio in neural networks, thereby facilitating cognitive processes such as attention, working memory, and cognitive flexibility [19, 20], although the precise mechanisms remain under investigation. Evidence from recent clinical neuromodulation studies supports the Left DLPFC as a key therapeutic target for improving emotion-related disturbances, reinforcing our rationale for applying left-sided stimulation in the present study [21]. We propose the following working model: tRNS over the Left DLPFC may hypothetically increase cortical excitability via stochastic resonance, which could enhance working memory, inhibitory control, and cognitive reappraisal. These processes might enable stronger top-down regulation of limbic regions (e.g., amygdala), potentially resulting in improved emotion regulation. Bilateral tRNS may cause inter-hemispheric interference, explaining its weaker effects. We hypothesized that unilateral (left) stimulation would more effectively enhance executive and emotion regulatory processes under induced dysregulation conditions.

## Materials and methods

### Procedure and participants

**Fig. 1:**
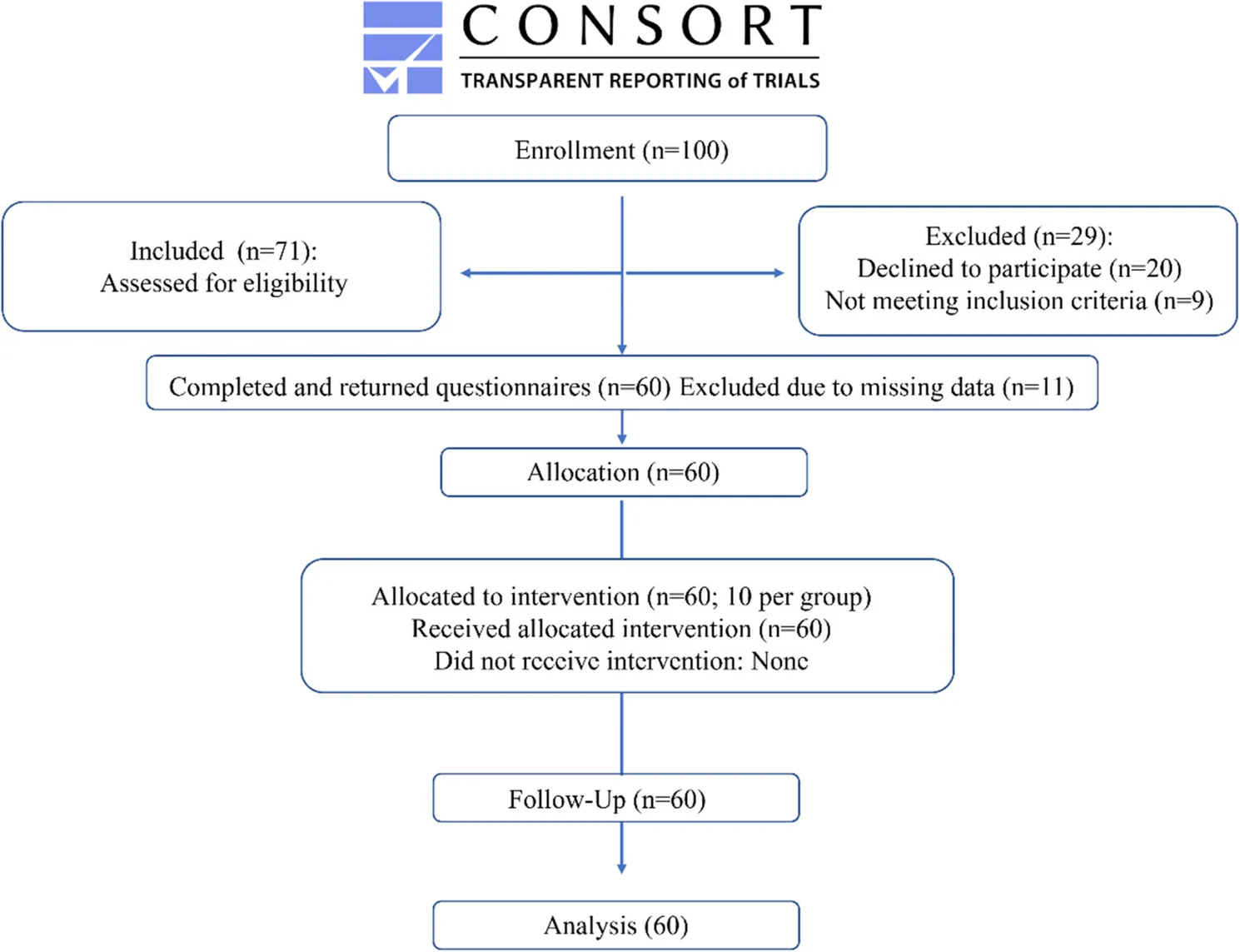
CONSORT diagram illustrating participant flow throughout the trial, from initial screening and randomization to final analysis, in accordance with the CONSORT 2010 guidelines

After obtaining approval from Baqiyatallah University of Medical Sciences, we used convenience sampling to recruit participants from Baqiyatallah University of Medical Sciences. Participants were recruited between February 2024 and August 2024, with follow-up continuing until December 2024. Eligibility criteria included enrollment as a male postgraduate student aged 28 years or older. The study was conducted in a dormitory, and postgraduate students were the most accessible participants. This selection was not based on age or educational rationale and may limit the generalizability of the findings. All participants were healthy adults with no self-reported history of psychiatric or neurological disorders. No structured clinical interview (e.g., SCID) was administered, as the study was not designed as a clinical analogue study. Instead, we aimed to induce a transient state of emotion dysregulation in a non-clinical sample to model socially-relevant emotion regulation challenges (e.g., repeated exposure to distressing news or social pressure). We distributed 71 survey packets, all of which were completed, resulting in a 100% response rate. Of the 71 completed surveys, 11 were excluded due to missing data. This study fully complies with the CONSORT standard guidelines for reporting clinical trials. The flow of participants through the trial is summarized in a CONSORT flow diagram, in accordance with CONSORT guidelines [22, 23] (Fig. 1). The pre-test (white) and post-test (brown) data collection yielded 60 usable surveys. Participants were randomly assigned to one of six groups using a computer-generated simple random allocation sequence without blocking or other restrictions. Randomization was performed to ensure unbiased allocation and comparable groups. No specific mechanism was used for allocation concealment. Participant baseline characteristics, including age and years of formal education, are summarized in Table 1. No significant differences were observed between the six groups, confirming successful randomization. The random allocation sequence was generated, participants were enrolled, and assignments to interventions were all carried out by MH. (1) Sham, (2) emotion dysregulation (Edys), (3) Left tRNS (L-tRNS), (4) left and right tRNS (LR-tRNS), (5) emotion dysregulation + left tRNS (Edys + L-tRNS), and (6) emotion dysregulation + left and right tRNS (Edys + LR-tRNS). To induce a transient state of emotional dysregulation, participants in the Edys group were exposed to 50 traumatic images. This manipulation was intended to temporarily disrupt emotion regulation in healthy adults. This study was conducted as a single-blind trial. Participants were blinded to their group assignments, while care providers and outcome assessors were aware of the allocation of the intervention. Efforts were made to maintain participant blinding throughout the study. This study was hypothesis-driven. In our initial pilot work, we attempted to use standardized international image sets (e.g., IAPS) to induce negative affect and emotional distress. However, these standardized images did not produce any detectable emotion dysregulation effect in our population. This is likely because the arousal and valence norms for these images are derived from Western populations and do not account for cultural differences in emotional evaluation, stimulus familiarity, or baseline emotional reactivity. Therefore, we developed a culturally relevant image set as follows: 50 participants were asked to select images from local news archives and social media content that personally evoked negative affect, distress, or emotional dysregulation. The 50 most frequently selected images were compiled into the final stimulus archive. We acknowledge that this image set is not validated in the same way as international standardized sets; rather, it was selected for ecological validity in our specific population. The images were presented automatically, with a presentation duration of 5-second per image and a 5-second inter-stimulus interval between each image. It is important to clarify that exposure to traumatic images primarily induces a transient state of negative affect and physiological arousal. In this study, we operationally define emotion dysregulation specifically as the significant elevation of DERS scores from pre- to post-induction. This operational definition allows us to quantify dysregulation as a state-like regulatory deficit in response to an acute emotional challenge, rather than assuming that the images themselves directly constitute dysregulation. Accordingly, we use the term dysregulation throughout the manuscript only in reference to this operationalized metric, and refer to the induction procedure itself as a negative affect induction or emotional distress induction. Participation was entirely voluntary, and in accordance with research ethics protocols, any participant could withdraw from the study at any time if they felt uncomfortable. We expected that left tRNS would improve emotion regulation and executive function more than bilateral stimulation, based on prior evidence linking Left DLPFC to regulatory and executive processes. All participants were screened through a brief clinical interview (not a structured diagnostic interview) to verify the absence of any obvious neurological or psychiatric disorders. Before enrollment, each participant received information regarding the study procedures and provided written informed consent in accordance with ethical research standards. All methods and analyses were conducted under the relevant guidelines and regulations, and the study protocol was approved by the Research Ethics Committee of Baqiyatallah University of Medical Sciences (IR.BMSU.BAQ.REC.1404.063). This trial was registered in the Iranian Registry of Clinical Trials (Trial registration identifier: IRCT20170417033485N2, registered on 2025/07/25).

**Table 1:** Baseline characteristics of participants across six intervention groups

Variable	Sham (*n* = 10)	Edys (*n* = 10)	L-tRNS (*n* = 10)	LR-tRNS (*n* = 10)	Edys + L-tRNS (*n* = 10)	Edys + LR-tRNS (*n* = 10)	*p*-value
Age (years)	30.00 ± 0.66	30.60 ± 1.57	30.90 ± 1.28	30.90 ± 0.99	30.00 ± 1.63	30.60 ± 0.84	0.35
Education (years)	19.50 ± 1.58	19.20 ± 1.54	20.10 ± 1.44	18.90 ± 1.44	19.20 ± 1.54	19.20 ± 1.54	0.59

Data are presented as mean ± SD. Age is reported in years. Education indicates the number of years of formal education completed. P-values were obtained from one-way ANOVA to compare differences across groups. No significant differences were observed between groups for age or education, indicating successful randomization

### Experimental timeline

**Fig. 2:**
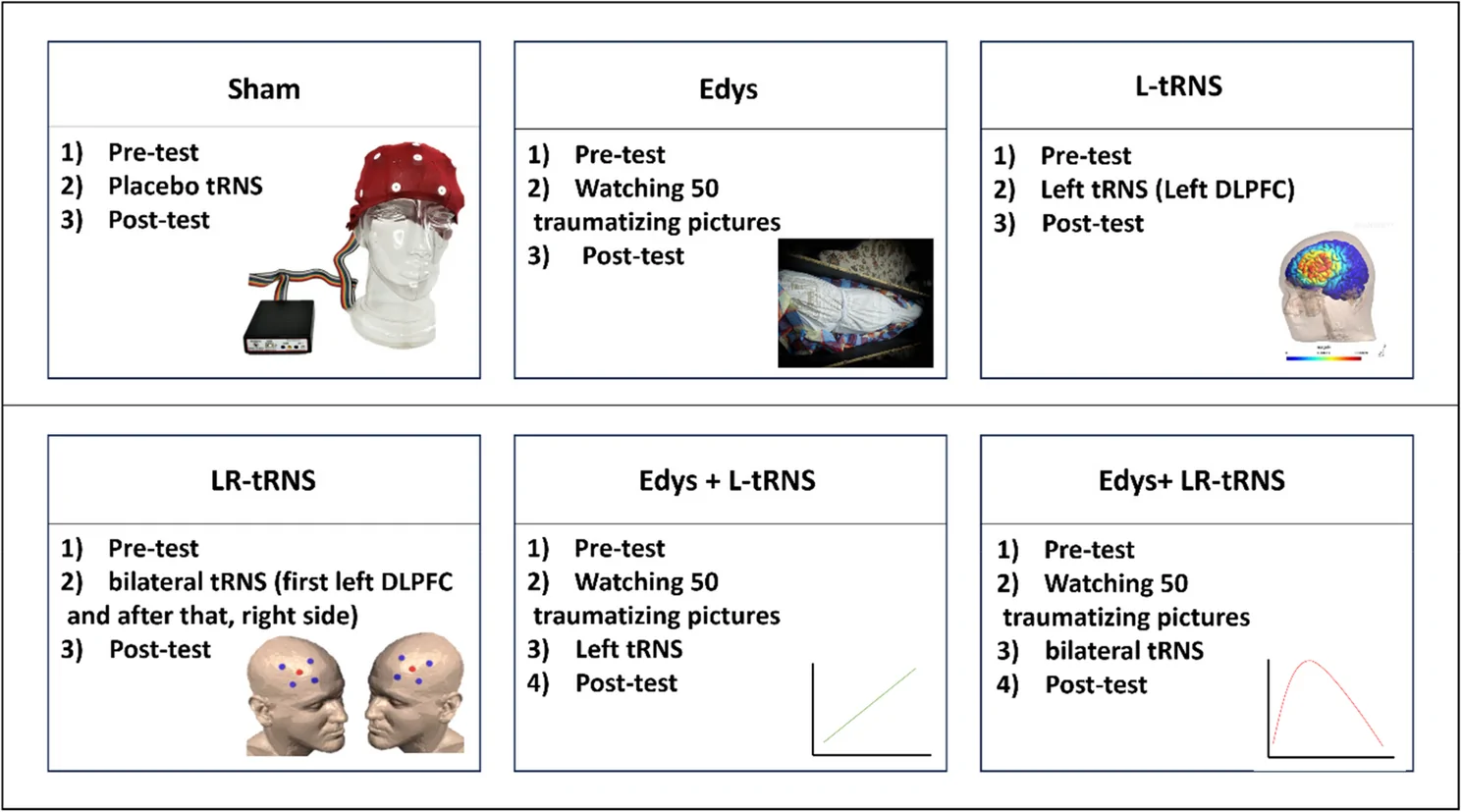
Schematic diagram of study groups

Baseline assessments (DERS, PASAT, ERQ, CERQ) were completed one week before the main experimental session. On the day of the experiment, participants either underwent emotion dysregulation induction (50 traumatic images, 5-second pause between images, ~ 12.5 min) or not, depending on their group allocation. Five minutes after the induction/rest period, participants in L-tRNS and Edys + L-tRNS groups, received 20 min of tRNS on the left DLPFC. For bilateral tRNS groups, stimulation consisted of 20 min on the left side, a 5-minute interval, followed by 20 min on the right side. Post-intervention assessments were administered within 5 min after the end of this period (Fig. 2).

### Measurements

To assess substance use, we included a question in the demographic form listing various illicit substances and asked participants if they had used any in the past 30 days. If they answered yes, they were asked to specify the frequency of use (number of times) during that period. In our sample, 0% of students reported using any illicit substances in the previous 30 days.

### Difficulties in emotion regulation scale (DERS)

The Difficulties in Emotion Regulation Scale (DERS) is a 36-item self-report questionnaire designed to assess emotional dysregulation across six dimensions: (1) non-acceptance of negative emotions, (2) difficulties in pursuing goal-directed behavior under distress, (3) limited access to effective emotion regulation strategies, (4) impulse control difficulties during emotional distress, (5) lack of emotional awareness, and (6) lack of emotional clarity. Participants rate each item on a 5-point Likert scale ranging from 1 (almost never) to 5 (almost always). The overall DERS score is calculated by summing the six subscale scores, resulting in a total score ranging from 36 to 180. Higher scores indicate greater difficulties with emotion regulation. Previous research has demonstrated strong internal consistency for the DERS, with Cronbach’s alpha values ranging from 0.77 to 0.96. Each subscale has also shown acceptable reliability, with Cronbach’s alpha exceeding 0.70. Moreover, the DERS has demonstrated satisfactory predictive and construct validity [24].

### Cognitive emotion regulation questionnaire (CERQ)

The Cognitive Emotion Regulation Questionnaire (CERQ) is a 36-item instrument designed to assess seven distinct cognitive strategies of emotion regulation, which can be classified as either maladaptive or adaptive. The maladaptive strategies include rumination, referring to the repetitive focus on negative emotions and their causes without moving toward resolution; catastrophizing, which reflects the tendency to magnify the seriousness of an event and expect the worst possible outcomes; self-blame, characterized by attributing the responsibility for negative experiences to oneself, often accompanied by feelings of guilt; and blaming others, which involves externalizing responsibility and holding others accountable for adverse events. In contrast, the adaptive strategies include acceptance, or the acknowledgment of reality without excessive resistance, thereby enabling more effective coping; positive reappraisal, which involves cognitively reframing adverse events in a constructive way to highlight growth or potential benefits; and refocus on planning, which emphasizes redirecting attention toward practical solutions and future actions to deal with difficulties. By capturing both maladaptive and adaptive approaches, the CERQ provides a comprehensive assessment of individual differences in cognitive strategies of emotion regulation. Scores are obtained from participants’ responses about their use of each strategy on a Likert scale (1 = almost never; 5 = almost always) [25].

### Emotion regulation questionnaire (ERQ)

The ERQ is a 10-item self-report measure designed to assess two aspects of emotion regulation: cognitive reappraisal (a strategy that involves changing the way one thinks about a situation to alter its emotional impact, considered adaptive), evaluated through six items, and expressive suppression (a strategy that involves inhibiting the outward expression of emotions, considered maladaptive), evaluated through four items. Each item is rated on a scale from 1 (strongly disagree) to 7 (strongly agree), resulting in a score range of 6–42 for cognitive reappraisal as a good strategy and 4–28 for expressive suppression as a bad strategy. Higher scores indicate more frequent use of reappraisal or suppression. Alpha reliabilities averaged 0.79 for cognitive reappraisal and 0.73 for expressive suppression [24].

### Paced auditory serial addition test (PASAT)

The PASAT was selected as a validated measure of working memory and sustained attention—prefrontal-dependent processes closely linked to emotion regulation. Impaired PASAT performance is associated with greater reliance on maladaptive strategies such as rumination, while improvements following DLPFC neuromodulation parallel enhanced affective regulation. Thus, the PASAT served as a behavioral index to examine whether tRNS-induced cognitive improvements correspond with enhanced emotion regulation. Participants sat in front of a monitor (distance: approximately 60 cm) and heard digits (1–9, duration of presentation: 433–567 ms) via in-ear headphones. The task was to add the current digit to the digit they heard before the last one (2-back). Results were indicated by pressing the corresponding key on a keyboard that was equipped with the response letters 2–18. Four key indices were derived from the PASAT. First, mental preparation was calculated as the mean reaction time for correct responses during the first 10 trials, reflecting the participant’s ability to establish an initial cognitive set. Second, sustained attention was calculated as the mean reaction time for correct responses across all 60 trials, reflecting the ability to maintain focus over time. Third, correct answers were recorded as the total number of correct responses out of 60 trials (accuracy score). Fourth, biological (working) age of the brain was estimated using the following algorithm: biological age = chronological age + (standardized residual of reaction time and accuracy) × 5, based on normative data from a healthy reference population. Higher biological age indicates poorer cognitive efficiency relative to age-matched norms [26]. Together, these indices offered a comprehensive profile of participants’ attentional control, processing speed, and cognitive resilience [27].

### Transcranial random noise stimulation (tRNS) protocol

**Fig. 3:**
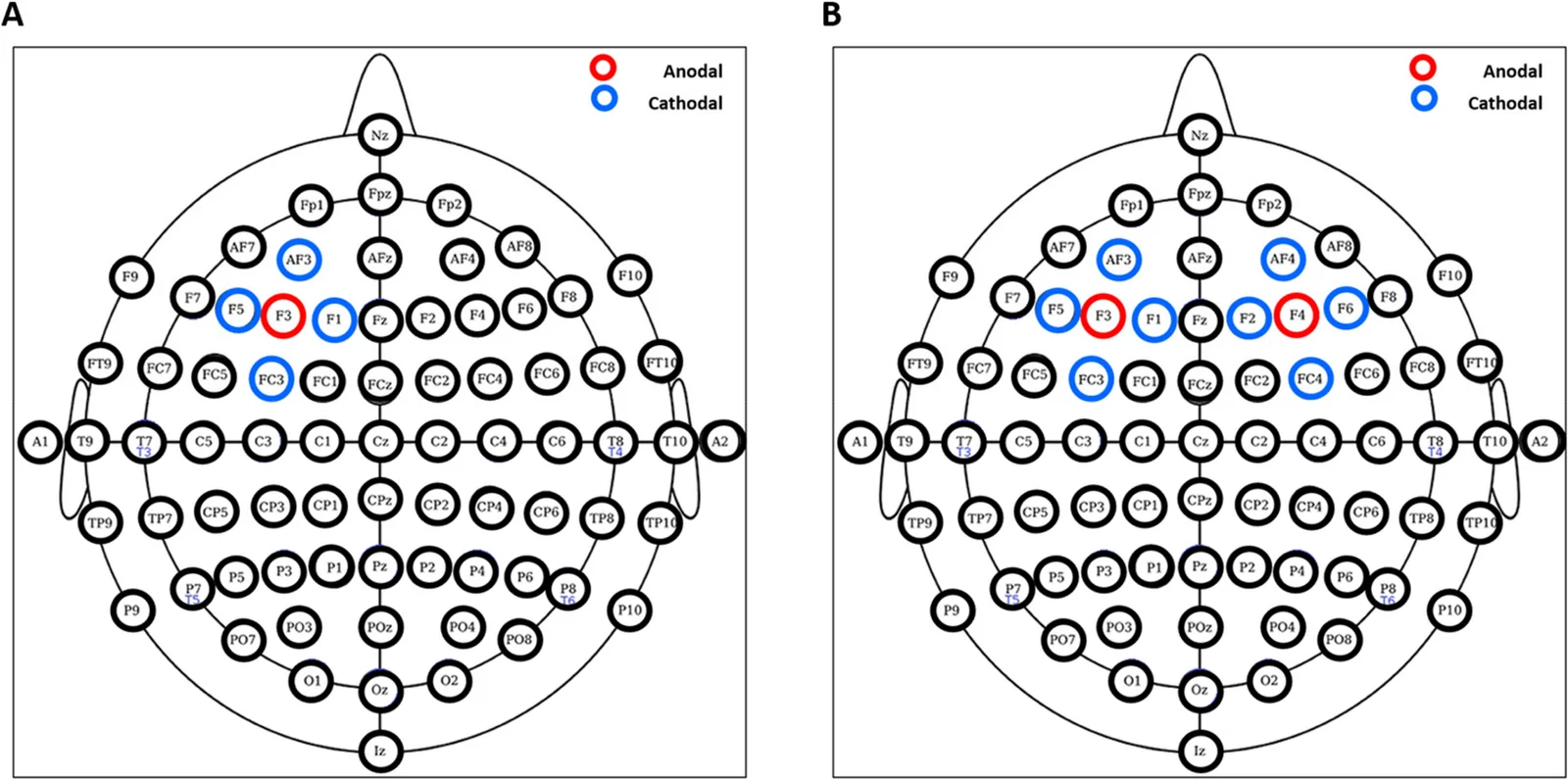
Schematic electrode montage for tRNS. **A** For unilateral left-sided stimulation, the anodal electrode was centered over F3, surrounded by four cathodal electrodes placed at F1, F5, AF3, and FC3. **B** For bilateral stimulation, an additional anodal electrode was placed over F4, surrounded by four cathodal electrodes at F2, F6, AF4, and FC4

**Fig. 4:**
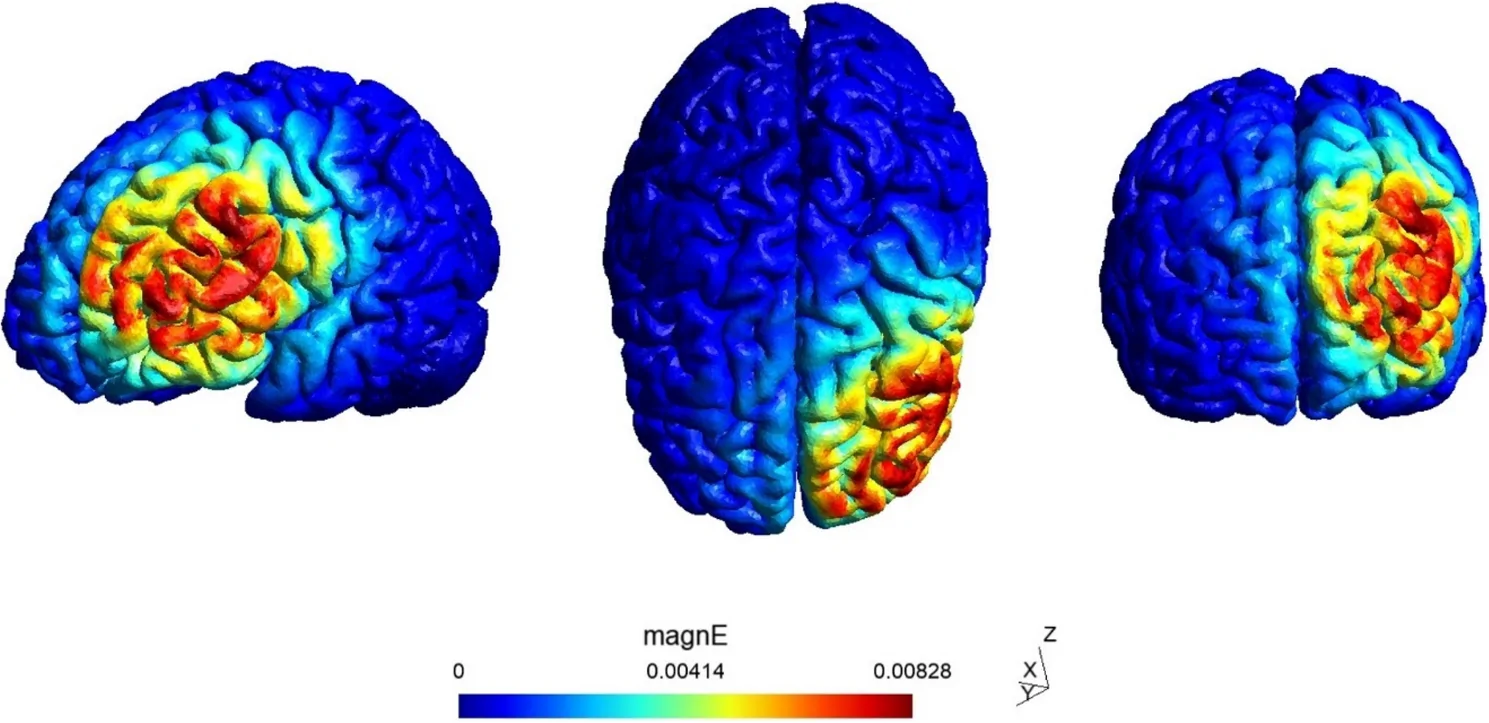
Distribution of the electrical field calculated using SimNIBS in the three views. The current intensity was set to 2 mA. Image generated by MH using SimNIBS software

Participants in the real tRNS groups received stimulation using a transcranial electric stimulator at 2 mA intensity for 20 min. The frequency range was set to 100–640 Hz (high-frequency random noise), with a Gaussian noise distribution (zero mean). For left tRNS, the central electrode was placed at F3, with four return electrodes arranged in a circle around F1, F5, AF3, and FC3 targeting the Left DLPFC. For bilateral tRNS, after stimulation of the Left DLPFC, the central electrode was placed at F4, with four return electrodes arranged around F2, F6, AF4, and FC4 targeting the right DLPFC (Fig. 3) [28]. Electrode placements were based on the international 10–20 system for EEG electrode localization. Electrodes were (1.5 × 1.5 cm) placed in saline-soaked sponges. The montage was designed to concentrate the electric field over the left and right DLPFC, respectively. Given the multi-electrode configuration and the proximity of the return electrodes to the central electrode, the field was expected to be primarily concentrated over the target frontal regions. The current was ramped up over the first 30 s and ramped down over the last 30 s of the 20-minute stimulation period. Sham stimulation followed the same electrode montage and ramp-up/ramp-down procedure (30 s ramp-up, followed by 30 s ramp-down), with no active current delivered. A detailed summary of all stimulation parameters, participant characteristics, and procedural details is provided in the RATES (Report Approval for Transcranial Electrical Stimulation) Table [29] (Table 2). The illustration modeling the flow of electrical current and examining the electric field distribution using SimNIBS is shown in Fig. 4 [30]. Sham participants received the same electrode setup with placebo stimulation, and all participants were blinded to the stimulation type.

**Table 2:** RATES checklist summary for tRNS parameters used in the present study

Domain	RATES Item	Description in the Present Study
Participants	Sample size (recruitment)	60 participants (10 per group), convenience sampling from postgraduate students
	Age (years), sex (M or F)	≥ 28 years, all male
	Inclusion/exclusion criteria	Inclusion: male postgraduate students aged 28+, no self-reported psychiatric/neurological disorders. Exclusion: incomplete data
	Caffeine, nicotine, alcohol, sleep hours	Not assessed
Stimulator	Stimulation device	Liv technology intelligence
	Sham condition	Yes (active sham protocol)
Electrodes	Positioning	Based on international 10–20 system; central over F3/F4; returns around F1/F5/AF3/FC3 and F2/F6/AF4/FC4
	Size	1.5 × 1.5 cm
	Material and interface	Electrodes in saline-soaked sponges
	Montage	Unilateral (left) and bilateral (sequential left and right)
	Inter-electrode distance	At least 6 cm
Current	Intensity (mA)	2 mA (base to peak, no DC offset)
	Waveform	Random noise with Gaussian distribution
	Frequency (Hz)	High-frequency noise: 100–640 Hz
	Duration	20 min
	Ramp-up/down (seconds)	30 s ramp-up, 30 s ramp-down
	Sham characteristics	30s ramp-up, 30s ramp-down
	Current density	calculated from 2 mA intensity and electrode area
Procedure	Study design	Randomized, single-blind clinical trial
	Blinding	Participants blinded; care providers and assessors aware of allocation
	Randomization method	Computer-generated simple random sequence; no allocation concealment
	Adverse event assessment	Not assessed
	Trial registration	Registered at Iranian Registry of Clinical Trials (IRCT20170417033485N2, registered on 2025/07/25)
	Ethical approval	Approved by Ethics Committee of Baqiyatallah University of Medical Sciences (IR.BMSU.BAQ.REC.1404.063)
	Informed consent	Obtained from all participants
	Data analysis	Two-way ANOVA, Bonferroni post-hoc, ANCOVA, change-score analyses

This table follows the RATES checklist for reporting transcranial electrical stimulation. Parameters are reported based on the tRNS protocol used in the present study

### Sample size determination

Based on prior literature [31] and preliminary data, a power analysis was conducted to determine an adequate sample size for detecting significant differences among groups. A minimum of 10 participants per group was included, which we acknowledge is modest. While an estimate of effect size was used from similar studies, this does not replace an a priori power calculation, and the results should be interpreted with caution.

### Statistical analysis

Results are presented as the mean ± standard error of the mean (Mean ± SEM). Data were analyzed using two-way ANOVA followed by Bonferroni’s post hoc test in GraphPad Prism 8. P values less than 0.05 were considered statistically significant. This analysis consisted of between-group comparisons of post-intervention DERS scores between groups, as well as within-group pre-post comparisons. Secondary outcomes included PASAT indices, ERQ, and CERQ scores. To control for potential baseline differences, we additionally performed ANCOVA with pre-test scores as covariates (Figs. 9, 10, 11 and 12). Exploratory analyses comprised comparisons involving the tRNS condition in two sides. All statistical assumptions (normality, homogeneity of variances) were verified before analysis. Given the modest sample size, caution is warranted when applying these findings to a broader context. Exact p-values and effect sizes for all major comparisons have been explicitly reported in the Results text to provide greater statistical transparency and interpretability.

## Results

### Effects of stimulation on parameters relating to DERS

**Fig. 5:**
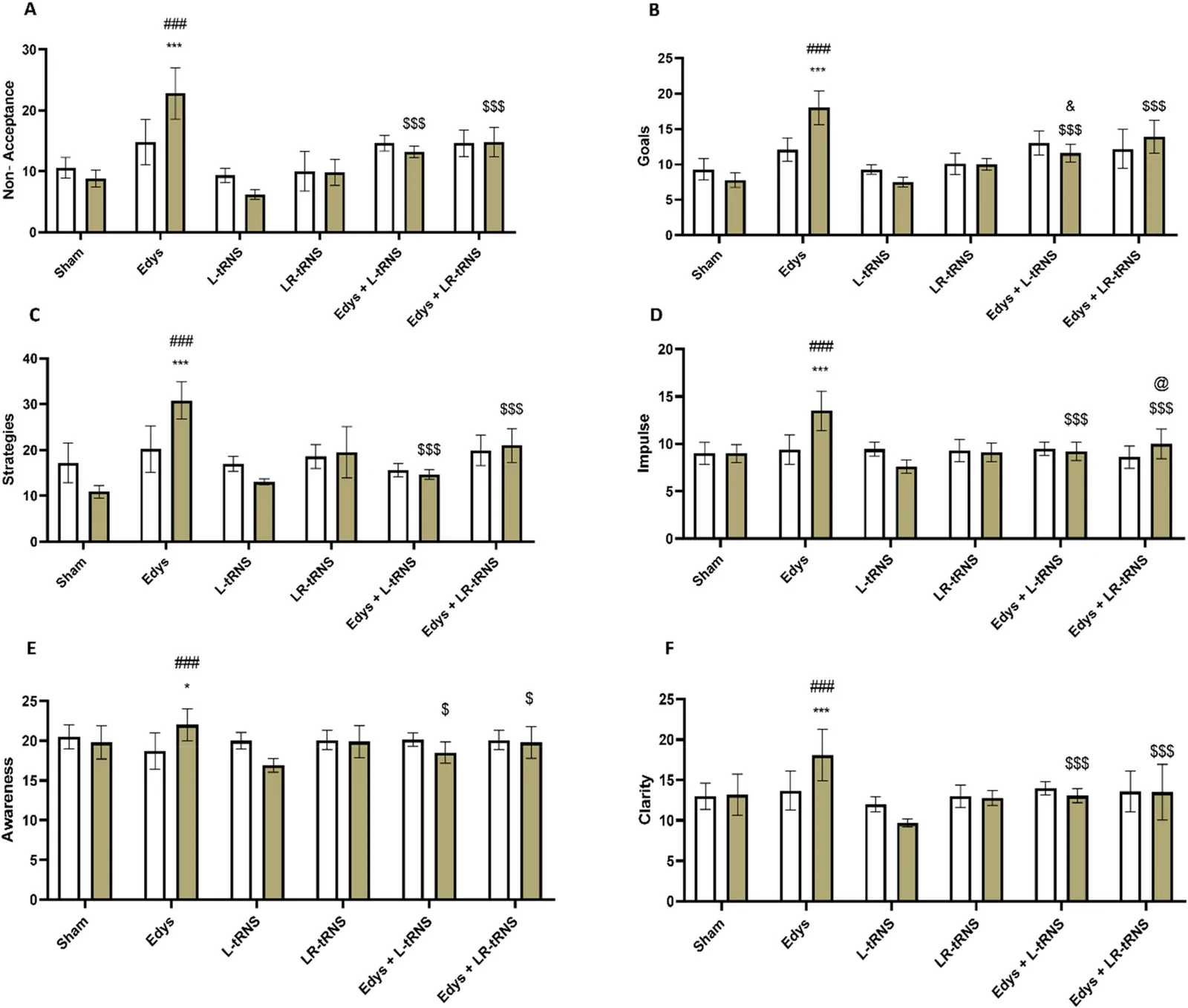
Changes in emotion regulation difficulties scales (DERS). Non-Acceptance (**A**), Goals (**B**), Strategies (**C**), Impulse (**D**), Awareness (**E**), and Clarity (**F**). Bars represent Mean ± SEM for each group: Sham, Edys, L-tRNS, LR-tRNS, Edys + L-tRNS, and Edys + LR-tRNS. White bars indicate pre-test scores, and brown bars indicate post-test scores. *p < 0.05 vs. post-test Sham. ***p < 0.001 vs. post-test Sham. ###*p* < 0.001 vs. pre-test Edys. $*p* < 0.05 vs. post-test Edys. $$$*p* < 0.001 vs. post-test Edys. & *p* < 0.05 vs. pre-test Edys + L-tRNS. @ *p* < 0.05 vs. pre-test Edys + LR-tRNS

Individuals in the Edys group showed significantly higher post-induction scores on the DERS subscales Non-Acceptance (Mean = 22.80, SEM = 1.33), Goals (Mean = 18.00, SEM = 0.74), Strategies (Mean = 30.80, SEM = 1.28), Impulse (Mean = 13.50, SEM = 0.65), and Clarity (Mean = 18.10, SEM = 1.00), compared with the post-test of Sham group and their own pre-test (Fig. 5A, D and F, *p* < 0.001) (partial η² = size effect [η²A = 0.39, η²B = 0.42, η²C = 0.32, η²D = 0.39, η²F = 0.28]). Scores on the Awareness subscale (Mean = 22.00, SEM = 0.63) were also significantly elevated in the Edys group, relative to post-test of Sham (Fig. 5E, *p* = 0.04) (partial η² = [η²E = 0.22]) and relative to own pre-test (Fig. 5E, *p* < 0.001) (partial η² = [η²E = 0.22]).

Post-test scores in the Edys + L-tRNS group were significantly lower on the DERS subscales Non-Acceptance (Mean = 13.20, SEM = 0.29), Goals (Mean = 11.60, SEM = 0.40), Strategies (Mean = 14.70, SEM = 0.33), Impulse (Mean = 9.22, SEM = 0.32), and Clarity (Mean = 13.10, SEM = 0.27), compared with the post-test of the Edys group (Fig. 5A, D and F, *p* < 0.001) (partial η² = size effect [η²A = 0.39, η²B = 0.42, η²C = 0.32, η²D = 0.39, η²F = 0.28]). Scores on the Awareness subscale (Mean = 18.50, SEM = 0.42) were also significantly reduced in the Edys + L-tRNS group, relative to the post-test of the Edys group (Fig. 5E, *p* = 0.03) (partial η² = [η²E = 0.22]).

In addition, post-test scores in the Edys + L-tRNS group were significantly lower on the Goals subscale (Mean = 11.60, SEM = 0.40) compared with their own pre-test (Fig. 5B, *p* = 0.03, [η²B = 0.42]).

Post-test scores in the Edys + LR-tRNS group were significantly higher on the Impulse subscales (Mean = 0.49, SEM = 0.49) compared with their own pre-test (Fig. 5D, *p* = 0.04, [η²D = 0.39]).

### Effects of stimulation on parameters relating to PASAT

**Fig. 6:**
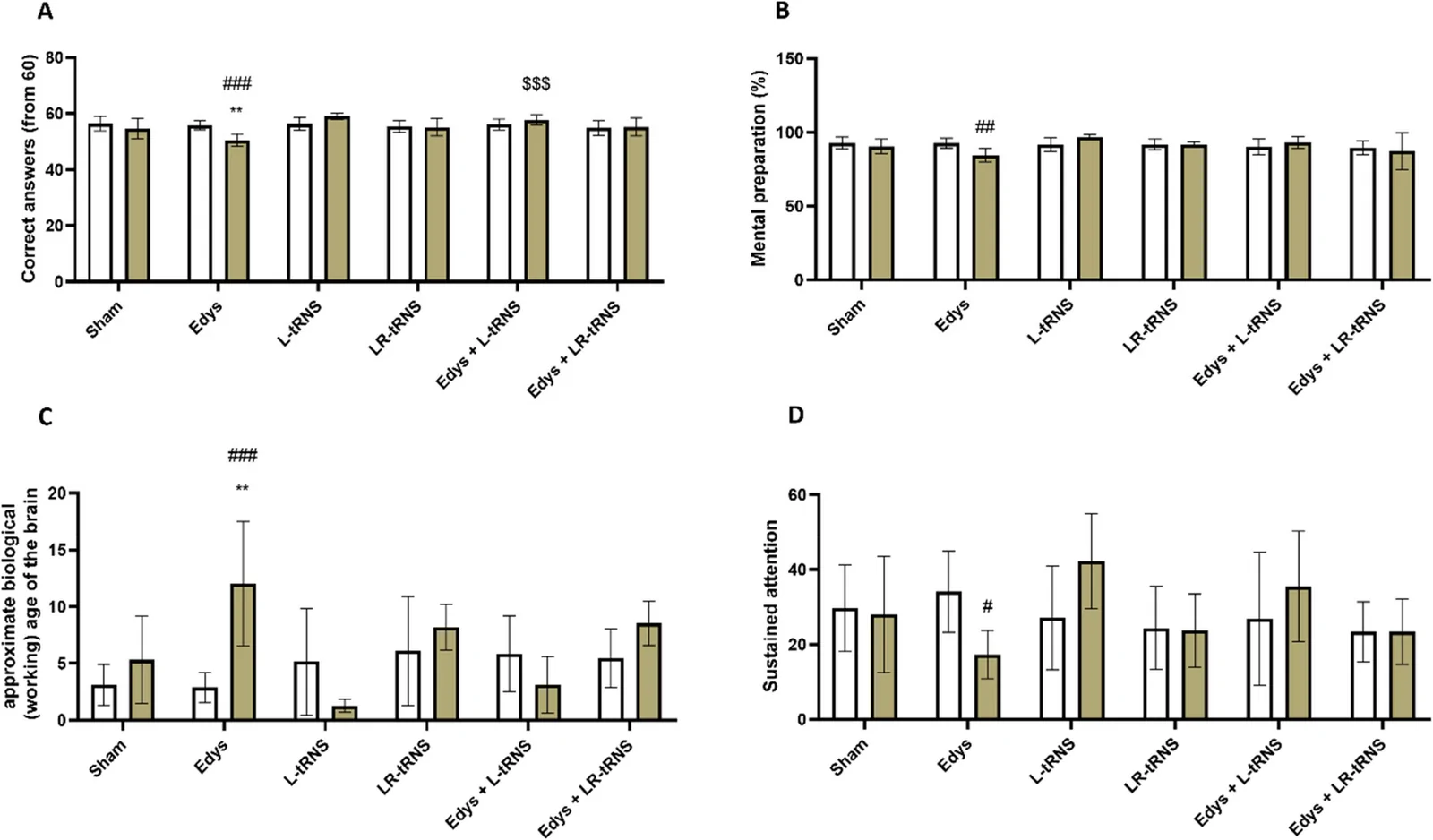
Changes in Paced Auditory Serial Addition Test (PASAT). Correct answer (**A**), Mental preparation (**B**), age of brain (**C**), and Sustained attention (**D**). Bars represent Mean ± SEM for each group: Sham, Edys, L-tRNS, LR-tRNS, Edys + L-tRNS, and Edys + LR-tRNS. White bars indicate pre-test scores, and brown bars indicate post-test scores. **p < 0.01 vs. post-test Sham. #*p* < 0.05 vs. pre-test Edys. ##*p* < 0.01 vs. pre-test Edys. ###*p* < 0.001 vs. pre-test Edys. $$$*p* < 0.001 vs. post-test Edys

Individuals in the Edys group showed significantly lower post-induction scores on Correct Answers (Mean = 50.44, SEM = 0.78), Mental Preparation (Mean = 84.61, SEM = 1.43), and Sustained Attention (Mean = 17.30, SEM = 2.03), and significantly higher scores on Biological (Working) Age of the Brain (Mean = 12.03, SEM = 1.72) compared with their own pre-test (Fig. 6A and C, *p* < 0.001, Fig. 6B, *p* < 0.01 and Fig. 6D, *p* = 0.04) (partial η² = size effect [η²A = 0.23, η²B = 0.15, η²C = 0.32, η²D = 0.99]) and significantly lower Correct Answers (Mean = 50.44, SEM = 0.78) and higher Biological (Working) Age of the Brain (Mean = 12.03, SEM = 1.72) compared with post-test of Sham group (Fig. 6A and C, *p* < 0.01) (partial η² = size effect [η²A = 0.23, η²B = 0.15]).

Post-test scores in the Edys + L-tRNS group were significantly higher on the Correct Answers subscale (Mean = 57.44, SEM = 0.60) compared with the post-test of the Edys group (Fig. 6A, *p* < 0.001, [η²A = 0.23]).

### Effects of stimulation on parameters relating to ERQ

**Fig. 7:**
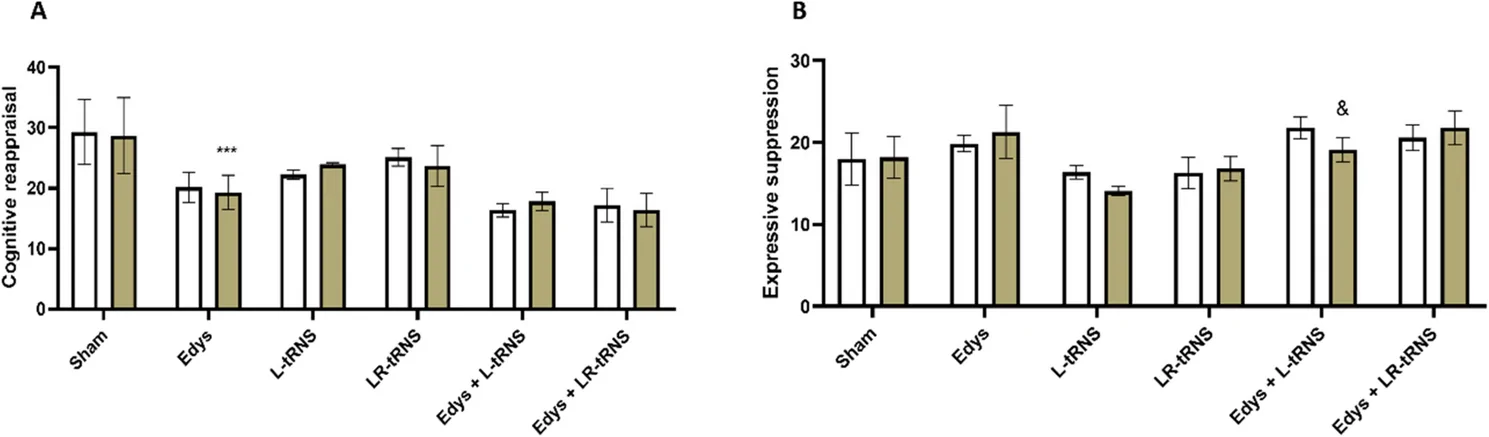
Changes in emotion regulation questionnaire (ERQ). Cognitive reappraisal (**A**) and Expressive suppression (**B**). Bars represent Mean ± SEM for each group: Sham, Edys, L-tRNS, LR-tRNS, Edys + L-tRNS, and Edys + LR-tRNS. White bars indicate pre-test scores, and brown bars indicate post-test scores. ***p < 0.001 vs. post-test Sham. & *p* < 0.05 vs. pre-test Edys + L-tRNS

Individuals in the Edys group showed significantly lower post-induction scores on Cognitive Reappraisal (Mean = 19.30, SEM = 0.89) compared with the post-test of the Sham group (Fig. 7A, *p* < 0.001, [η²A = 0.03]).

Additionally, scores in the Edys + L-tRNS group were significantly lower on Expressive Suppression (Mean = 19.11, SEM = 0.48) compared with their own pre-test (Fig. 7B, *p* = 0.04, [η²B = 0.15]).

### Effects of stimulation on parameters relating to CERQ

**Fig. 8:**
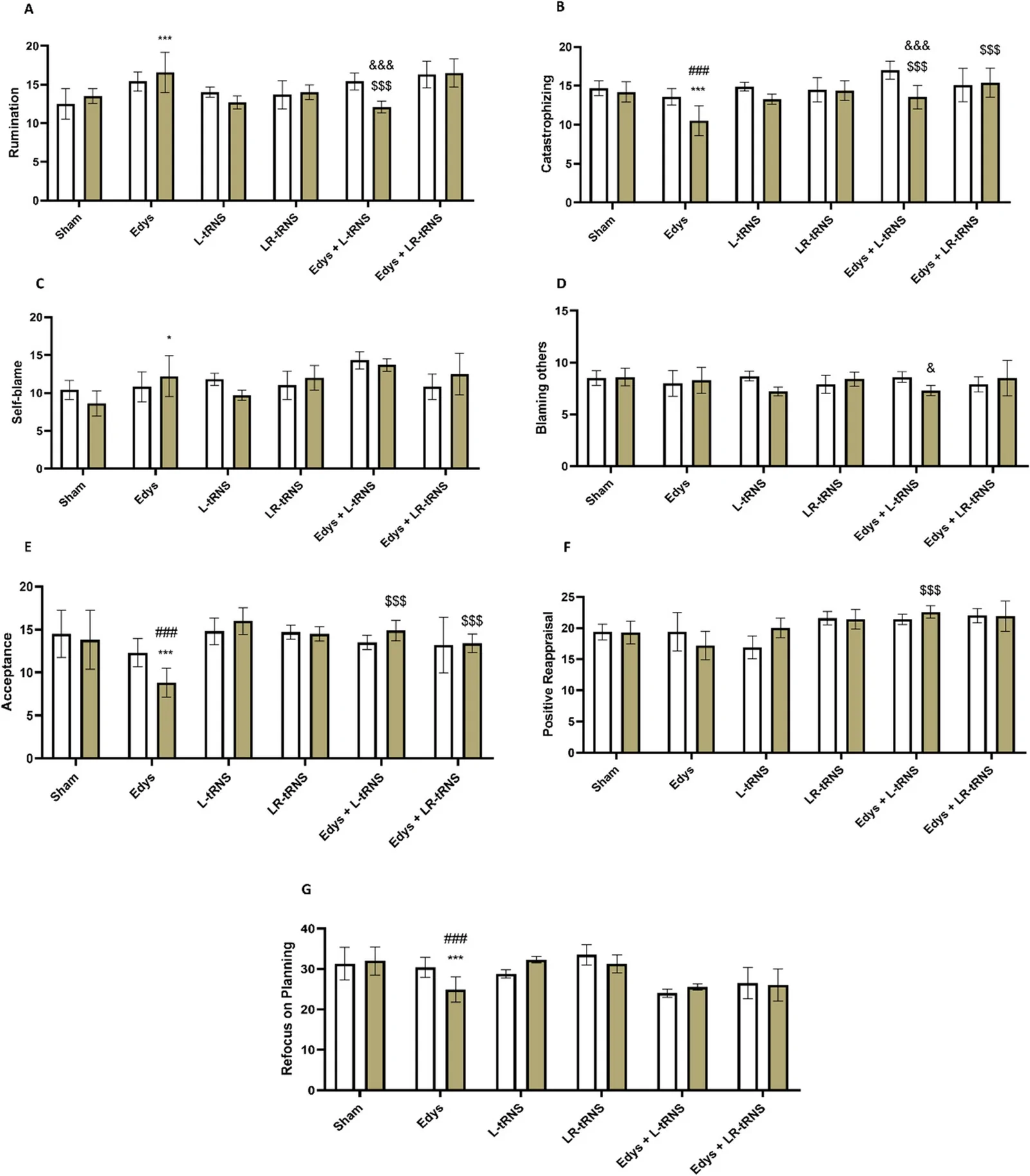
Changes in cognitive emotion regulation questionnaire (CERQ)**.** Rumination (**A**), Catastrophizing (**B**), Self-blame (**C**), Blame others (**D**), Acceptance (**E**), Positive reappraisal (**F**), and refocus on planning (**G**). Bars represent Mean ± SEM for each group: Sham, Edys, L-tRNS, LR-tRNS, Edys + L-tRNS, and Edys + LR-tRNS. White bars indicate pre-test scores, and brown bars indicate post-test scores. *p < 0.05 vs. post-test Sham. ***p < 0.001 vs. post-test Sham. ###*p* < 0.001 vs. pre-test Edys. $$$*p* < 0.001 vs. post-test Edys. &*p* < 0.05 vs. pre-test Edys + L-tRNS. &&& *p* < 0.001 vs. pre-test Edys + L-tRNS

Individuals in the Edys group showed significantly lower post-induction scores on the CERQ subscales Catastrophizing (M = 10.50, SE = 0.60), Acceptance (M = 8.80, SE = 0.53), and Refocus on Planning (M = 24.90, SE = 0.98), and significantly higher scores on Rumination (M = 16.60, SE = 0.81) and Self-blame (M = 12.20, SE = 0.85), compared with the Sham post-test (Fig. 8A, B, E and G, *p* < 0.001 and Fig. 8C, *p* = 0.01) (partial η² = size effect [η²A = 0.22, η²B = 0.22, η²C = 0.17, η²E = 0.16, η²G = 0.23]).

Scores on the Catastrophizing (Mean = 10.50, SEM = 0.60), Acceptance (Mean = 8.80, SEM = 0.53) and Refocus on Planning subscale (Mean = 24.90, SEM = 0.98) were also significantly lowered in the Edys group, relative to their own pre-test (Fig. 8B, E and G, *p* < 0.001) (partial η² = size effect [η²B = 0.22, η²E = 0.16, η²G = 0.23]).

Post-test scores in the Edys + L-tRNS group were significantly lower on Rumination (M = 12.11, SE = 0.26) and significantly higher on Catastrophizing (M = 13.56, SE = 0.50), Acceptance (M = 14.90, SE = 0.37), and Positive Reappraisal (M = 22.60, SE = 0.30), compared with the post-test of the Edys group (Fig. 8A, B and E, and 8F, *p* < 0.001) (partial η² = size effect [η²A = 0.22, η²B = 0.22, η²E = 0.16, η²F = 0.30]).

In addition, scores in the Edys + L-tRNS group were significantly lower on Rumination (M = 12.11, SE = 0.26), Catastrophizing (M = 13.56, SE = 0.50), and Blaming Others (M = 7.30, SE = 0.15) compared with their own pre-test (Fig. 8A and B, *p* < 0.001 and Fig. 8D, *p* = 0.04) (partial η² = size effect [η²A = 0.22, η²B = 0.22, η²D = 0.19]).

### Changes in pre-post scores across groups

**Fig. 9:**
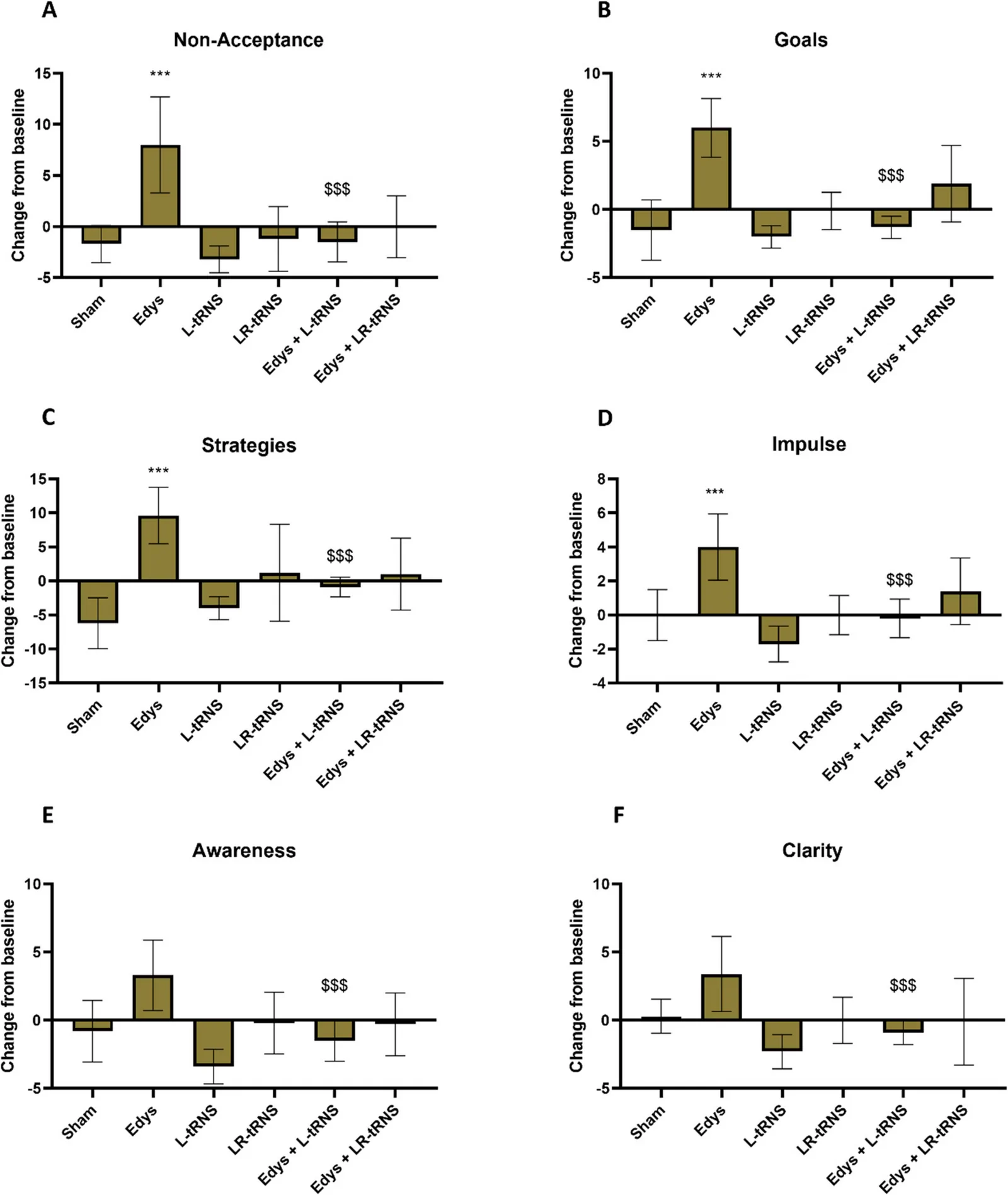
Post–pre change scores in DERS subscales across groups. Non-Acceptance (**A**), Goals (**B**), Strategies (**C**), Impulse (**D**), Awareness (**E**), and Clarity (**F**). Bars represent Mean ± SEM for each group: Sham, Edys, L-tRNS, LR-tRNS, Edys + L-tRNS, and Edys + LR-tRNS. ***p < 0.001 vs. Sham group. $$$*p* < 0.001 vs. Edys group

Significant differences between the Edys group and the Sham group were observed for Non-Acceptance (M = 8.00, SE = 1.49), Goals (M = 6.00, SE = 0.68), Strategies (M = 9.60, SE = 1.31), Impulse (M = 4.00, SE = 0.61) (Fig. 9A, B, C, D, *p* < 0.001) (partial η² = size effect [η²A = 2.72, η²B = 3.42, η²C = 4.00, η²D = 2.31]). Significant differences between the Edys + L-tRNS group and the Edys group were observed for Non-Acceptance (M = -1.50, SE = 0.61), Goals (M = -1.30, SE = 0.26), Strategies (M = -0.90, SE = 0.45), Impulse (M = -0.20, SE = 0.35), Awareness (M = -1.50, SE = 0.47), and Clarity (M = -0.90, SE = 0.27) (Fig. 9A, B, C, D, E, and F, *p* < 0.001) (partial η² = size effect [η²A = 2.63, η²B = 4.47, η²C = 3.39, η²D = 2.64, η²E = 2.27, η²F = 2.10]).

**Fig. 10:**
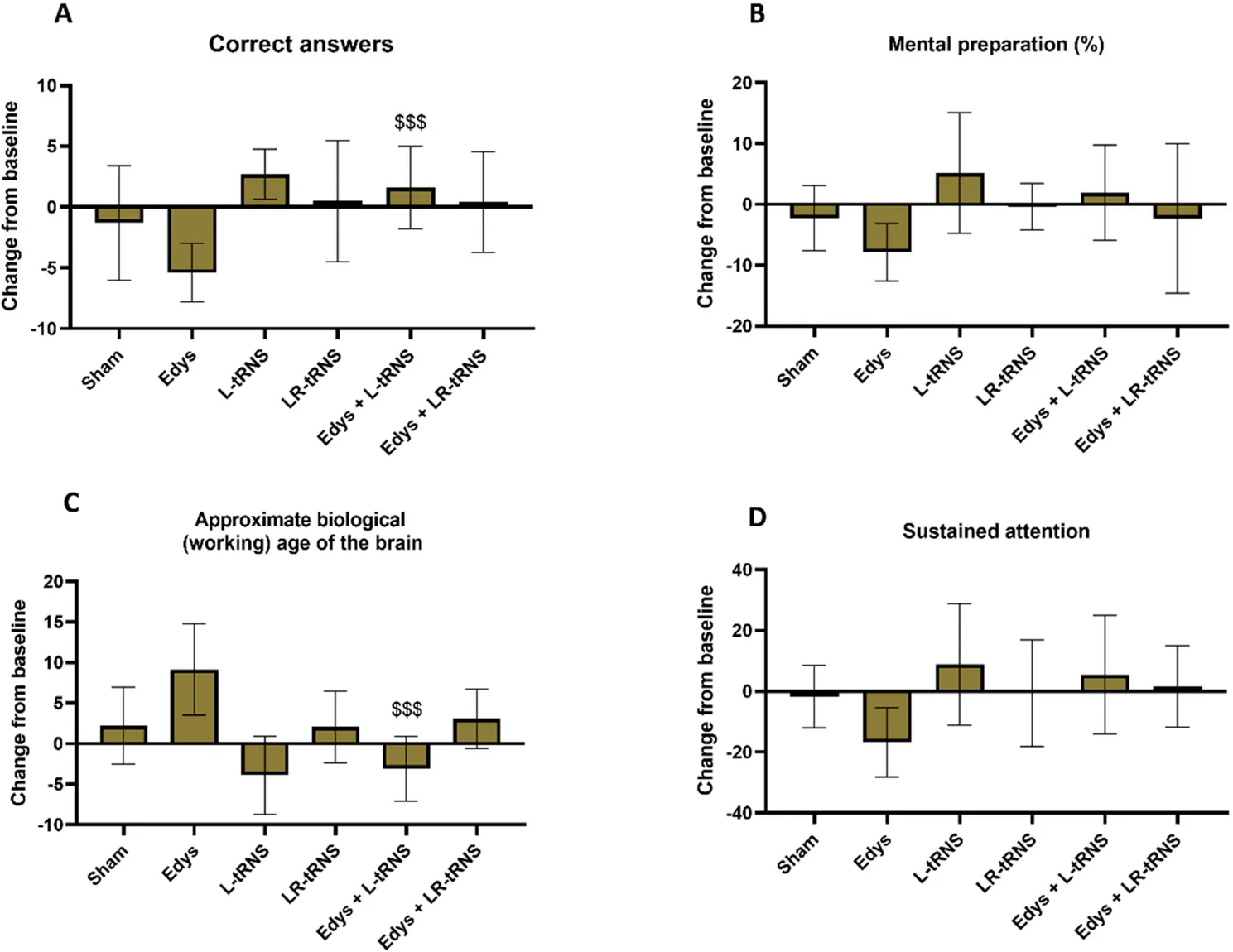
Post–pre change scores in PASAT subscales across groups. Correct answers (**A**), Mental preparation (**B**), biological age of brain (**C**), and Sustained attention (**D**). Bars represent Mean ± SEM for each group: Sham, Edys, L-tRNS, LR-tRNS, Edys + L-tRNS, and Edys + LR-tRNS. $$$*p* < 0.001 vs. Edys group

Significant differences between the Edys + L-tRNS group and the Edys group were observed for Correct Answers (M = 1.60, SE = 1.07) and biological age of the brain (M = -3.10, SE = 1.26) (Fig. 10A and C, *p* < 0.001) (partial η² = size effect [η²A = 2.37, η²C = 2.50]).

Significant differences between the Edys + L-tRNS group and the Edys group were observed for Rumination (M = -2.40, SE = 0.93), Acceptance (M = 1.40, SE = 0.42), and Positive Reappraisal (M = 1.20, SE = 0.42), (Fig. 12A and E, *p* < 0.001 and Fig. 12F, *p* < 0.01) (partial η² = size effect [η²A = 2.37, η²E = 2.67, η²F = 1.90]).

## Discussion

The present study aimed to investigate the effect of tRNS applied to the DLPFC, either on the left side or bilaterally, on improving emotion regulation in healthy subjects in whom emotional dysregulation was induced through exposure to socially traumatic images. This study indicated that individuals with emotion dysregulation had significantly higher total scores on the Non-Acceptance, Goals, Strategies, Impulse, Awareness, and Clarity subscales of the DERS (Fig. 5). Induced emotion dysregulation also resulted in significantly lower total scores on the Correct answers, Mental preparation, and Sustain attention and higher total scores on the biological age of the brain (Fig. 6). These findings, along with significant increases in Rumination, Self-blame, and significant decreases in Acceptance and Cognitive reappraisal, confirm that individuals who viewed 50 traumatic images were in an induced state of emotion dysregulation (Figs. 7 and 8). The application of tRNS to the Left DLPFC significantly improved total DERS scores compared to post-test scores in the Edys group and increased Correct answers on the PASAT, Acceptance and Positive reappraisal on CERQ scores compared to post-test scores in the Edys group. Consistent with our operational framework, the significant pre-to-post increase in DERS scores within the Edys group confirms that the induction procedure successfully created a transient regulatory challenge—indexed by perceived difficulties in emotion regulation—rather than merely inducing negative mood. This distinction is critical, as it positions our findings within the context of regulatory system strain, not simply affective reactivity.

**Fig. 11:**
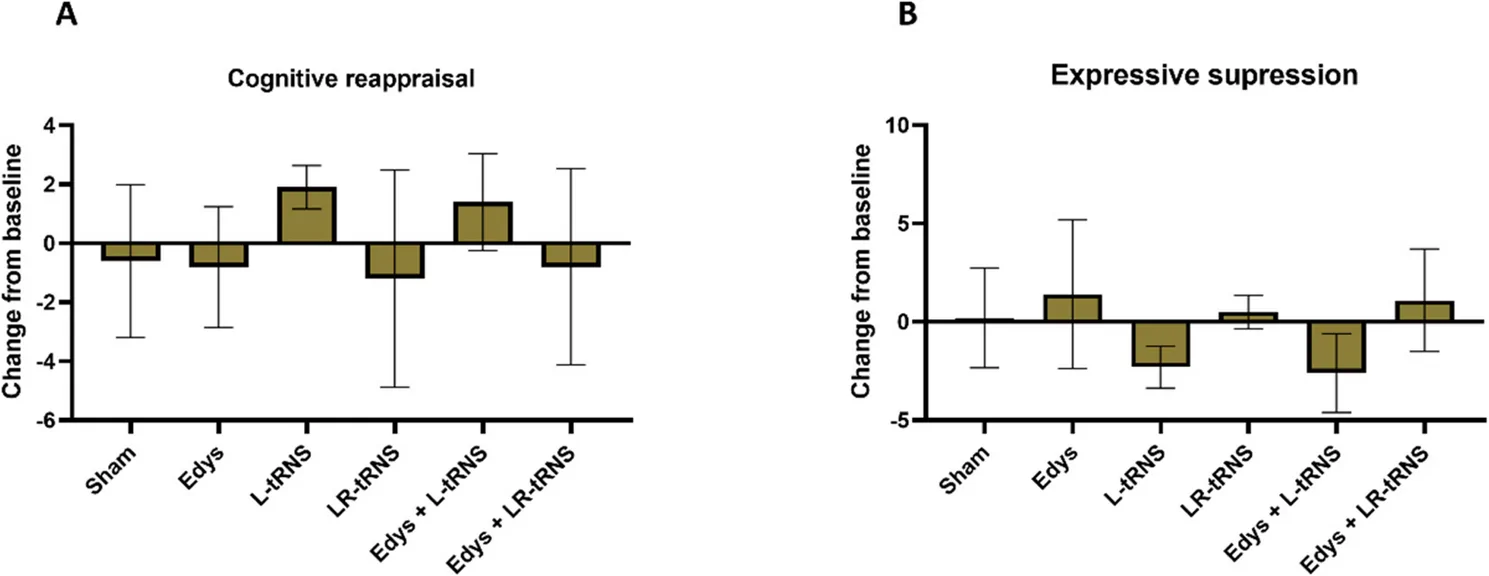
Post–pre change scores in ERQ subscales across groups. Cognitive reappraisal (**A**) and Expressive suppression (**B**). Bars represent Mean ± SEM for each group: Sham, Edys, L-tRNS, LR-tRNS, Edys + L-tRNS, and Edys + LR-tRNS

**Fig. 12:**
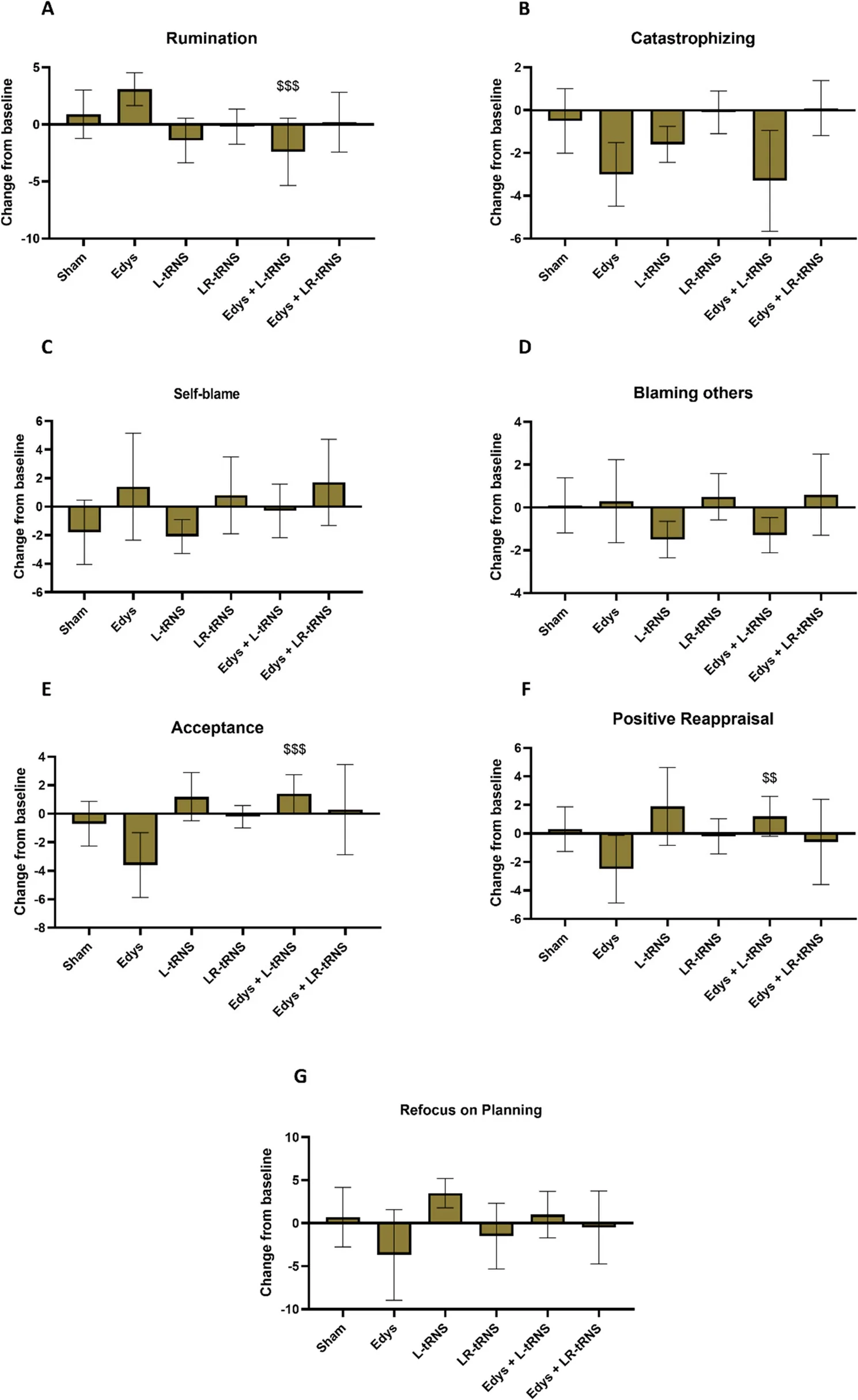
Post–pre change scores in CERQ subscales across groups. Rumination (**A**), Catastrophizing (**B**), Self-blame (**C**), Blame others (**D**), Acceptance (**E**), Positive reappraisal (**F**), and refocus on planning (**G**). Bars represent Mean ± SEM for each group: Sham, Edys, L-tRNS, LR-tRNS, Edys + L-tRNS, and Edys + LR-tRNS. $$*p* < 0.01 vs. Edys group. $$$*p* < 0.001 vs. Edys group

On the other hand, use of tRNS on Left DLPFC caused a significant decrease in score of Expressive suppression relating to ERQ and score of Rumination, Catastrophizing, and Blaming others relating to CERQ than pre-test of Edys + L-tRNS. It is concluded that likely Expressive suppression, Rumination, Catastrophizing, and Blaming others as bad indices in neuronal processing were under ameliorating effect of tRNS stimulation, which didn’t occur with the LR-tRNS group. As shown in Figs. 9, 10, 11 and 12, post–pre change scores were analyzed. In Fig. 9, significant differences were observed between Edys group and Sham group for Non-Acceptance, Goals, Strategies, Impulse and significant differences were observed between Edys + L-tRNS group and Edys group for Non-Acceptance, Goals, Strategies, Impulse, Awareness, and Clarity. In Fig. 10, significant differences were observed between Edys + L-tRNS group and Edys group for Correct answers and biological age of the brain. In Fig. 12, significant differences were observed between Edys + L-tRNS group and Edys group for Rumination, Acceptance, and Positive reappraisal. Covariance analysis revealed that Catastrophizing and Refocus on planning which had been affected by the tRNS in Edys + L-tRNS, were unreliable and could not be meaningfully interpreted, as they exhibited no significant differences across experimental groups. In contrast, the analysis revealed that biological age of the brain, in Edys + L-tRNS group, showed significant changes compared to the Edys group—an effect that had not been observed in the initial analysis.

The data reveal that the application of bilateral tRNS resulted in a marked enhancement in the Impulse score associated with the DERS when compared to the pre-test results of Edys + LR-tRNS. This finding implies that bilateral tRNS could potentially exert adverse effects on the DLPFC.

This form of stimulation has been investigated in various cognitive tasks, including the PASAT test, which is widely used to assess cognitive flexibility, working memory, and attention. In the present study, the ameliorating effects of tRNS in the L-tRNS group were confirmed through the analysis of PASAT test results.

Previous studies reported that tRNS has been shown to improve executive function, including working memory, impulse control, and processing speed, particularly in studies on attention-deficit/hyperactivity disorder [32, 33]. This effect was particularly noticeable when tRNS was applied to the prefrontal cortex, a region known to play a central role in executive functions and cognitive flexibility, which are critical in the PASAT test. In this study, people in Edys + L-tRNS groups were able to switch between tasks more efficiently and showed enhanced accuracy, suggesting that tRNS over the left DLPFC may enhance cognitive flexibility and executive function, although this interpretation remains speculative without direct neural evidence [34].

It is stated that, although the capacity for cognitive emotion regulation is universal, individuals differ in the thoughts they use to regulate their emotions in response to life experiences. This explains why some people have no difficulty regulating their emotions, while others do [35]. In this study, we used seven Garnefski’s criteria that were divided into two groups. First, adaptive strategies include Acceptance (expression of beliefs of acceptance related to the event), Refocus on Planning (concentration on the phases to be taken to cope effectively with the experience), and Positive Reappraisal (giving positive meaning to the experience and making positive deductions). Secondly, non-adaptive strategies include Self-blame (assigning responsibility for the experience to oneself), blaming others (assigning responsibility for the experience to others), rumination (obsessive thinking about the feelings associated with the experience), and Catastrophizing (emphasizing the experience’s negative aspects). In recent research, strong relationships were found between the use of these poor coping strategies and impaired emotion dysregulation. In general, results suggest that people who use maladaptive cognitive styles, such as rumination, catastrophizing, and self-blame, may be more vulnerable to emotional problems than those who use adaptive strategies, such as positive reappraisal [35].

As shown in our data, after recruiting tRNS on the left side of the DLPFC, unlike bilateral tRNS, various strategies indicating a higher level of cognitive processing that a person can adopt to cope with distressing situations during emotion dysregulation—such as Acceptance and Positive Reappraisal as measured by the CERQ—had higher and improved scores. Conversely, maladaptive strategies indicating a lower level of cognitive processing for confronting distressing situations during emotion dysregulation—such as rumination—had lower scores. Based on a previous study, which was designed by Kazemi et al., [36] individual differences should be considered when interpreting the observed effects of tRNS on adaptive and maladaptive emotion regulation.

By applying tRNS over Left DLPFC, it could probably promote the reorganization of neural circuits in the prefrontal cortex, leading to more efficient task switching, better attention regulation, and improved working memory—key components of the PASAT test. Additionally, tRNS over Left DLPFC might increase neural plasticity, facilitating faster and more adaptive responses to new tasks [19, 37].

Neuroscience studies have shown that activity in frontal regions of the brain, including the DLPFC, plays an important role in cognition and emotion regulation [38]. Since the PFC is involved in cognitive and emotional responses, PFC networks, including the DLPFC and vlPFC, play an important role in modulating emotional responses. It has been shown that when individuals are taught to regulate their emotional responses to emotional stimuli (i.e., reappraisal), activity in the Left DLPFC increases [19]. On the other hand, Ambrus et al. (2011) stated that application of tRNS to the Right DLPFC impaired categorical learning in a prototype distortion task [39]. The data disclose that recruitment of bilateral tRNS is associated with impaired emotion regulation, mood, and deficits in emotional attention regulation, while increased activity in the Left DLPFC has merely been associated with emotion efficiency and emotional attention regulation. It is possible that stimulation over the Left DLPFC enhanced activity in this region, contributing to improved emotion regulation, whereas bilateral stimulation could introduce competing activity between hemispheres, potentially reducing regulatory efficiency [40]. On the other hand, the observed increase in some DERS subscales following bilateral tRNS suggests several mechanisms could underlie this effect. First, simultaneous stimulation of both hemispheres may disrupt lateralized control functions, as the left and Right DLPFC are involved in different aspects of emotion regulation [41]. Second, bilateral stimulation might alter the balance of network connectivity between the DLPFC and subcortical regions such as the amygdala and hippocampus, reducing the efficacy of top-down regulatory processes. Third, it is possible that each hemisphere preferentially engages different regulatory strategies, so concurrent activation may induce conflicting processing, thereby impairing overall regulation. Future research is required to clarify these mechanisms and determine the optimal stimulation parameters for enhancing emotion regulation. These interpretations remain speculative and should be confirmed in future studies using neuroimaging or electrophysiological techniques.

Although our study used transiently induced emotion dysregulation in healthy adults, prior research has shown that deficits in emotion regulation mediated by various psychological distress, highlighting the central role of regulatory processes [42].

However, these interpretations regarding neural mechanisms remain speculative and should be confirmed using neuroimaging or other direct measures of brain activity.

The modest sample size (*n* = 10 per group) and division into six groups – while necessary to answer multiple experimental questions – significantly limit statistical power and the robustness of between-group comparisons. The small sample size also increases the risk of both Type I (false positive) and Type II (false negative) errors. Therefore, our findings should be interpreted as exploratory and preliminary, and all between-group differences require replication in larger samples. The subgroup analyses (comparisons among six groups) are particularly underpowered, and we have tempered our conclusions accordingly. Although all groups were equivalent at pre-test and ANCOVA adjusted for baseline measures, floor/ceiling effects inherent to the induction procedure may affect comparisons between induced and non-induced groups. The study population consists exclusively of male postgraduate students, which significantly limits generalizability to females, other age groups, and broader populations. Sex differences in emotion regulation and responsiveness to neuromodulation are well documented; therefore, our findings may not extend to females. Furthermore, the absence of neuroimaging data prevents direct examination of the proposed neural mechanisms, leaving our working model speculative. We also did not include additional computerized tasks or physiological measures beyond the PASAT and questionnaires.

The single-blind design, lack of allocation concealment, and the same researcher performing multiple roles may have introduced bias in self-report measures. Although the PASAT is less susceptible to this bias, subjective measures should be interpreted with caution. Future studies should adopt double-blind designs with independent researchers.

Another limitation of this study is the absence of structured clinical interviews to rule out undiagnosed psychiatric conditions. However, our goal was not to model a clinical disorder but to investigate tRNS effects on experimentally-induced emotion dysregulation in healthy individuals. Future studies should replicate these findings in clinical populations.

## Conclusion

It is important to underscore that this study models an acute emotional challenge in healthy individuals, operationalized as a transient state-level regulatory deficit indexed by DERS change scores. This does not equate to clinical emotion dysregulation, and all interpretations are confined to this experimental framework. In this study, the results obtained before and after the test during the application of tRNS revealed that stimulation over the Left DLPFC effectively improved transiently induced emotional dysregulation in healthy participants. Following stimulation, participants were better able to adopt adaptive strategies such as acceptance, positive reappraisal, and refocusing on planning, while reducing maladaptive strategies like rumination when confronting stress. These findings are preliminary and hypothesis-generating, derived from an experimental model of acute emotional challenge rather than a clinical sample. Accordingly, they do not directly generalize to clinical populations, but offer preliminary behavioral insights into how neuromodulation may facilitate regulatory processes under emotionally demanding conditions. Given the small sample size and exploratory nature of this study, all findings require replication in larger, confirmatory studies before any definitive conclusions can be drawn. Additionally, due to procedural differences in stimulation duration between unilateral and bilateral conditions, interpretations regarding bilateral tRNS should be made with particular caution. 

## Supplementary Information

Supplementary Material 1.

Supplementary Material 2.

## Data Availability

Data will be available upon request.
