# Supplementary material for: Transcranial random noise stimulation (tRNS) over the left dorsolateral prefrontal cortex ameliorates emotion dysregulation and executive function: a single-blind, randomized, sham-controlled clinical trial
Source: BMC Psychol. 2026 Jul 17;14:1467. doi: 10.1186/s40359-026-05214-w (PMC13656383; doi:10.1186/s40359-026-05214-w)
Supplement: Supplementary file 1 [file 40359_2026_5214_MOESM1_ESM.pdf]

Investigating the effectiveness of transcranial random noise stimulation on the dorsolateral prefrontal cortex on emotion dysregulation and information processing speed

✔ Approved

Trial Id

84451

IRCT Id

IRCT20170417033485N2

Registration date

2025-07-25, 1404/05/03

Registration date

2025-07-25, 1404/05/03

Membership number

33485

If you have a feedback for the referee or an explaination regarding referee messages/entered data, please leave it here.

با سلام و عرض ادب و احترام

با تشکر از زحمات داور/داوران محترم

اصلاحات تقاضا شده توسط داور/داوران محترم طرح "بررسی اثربخشی تحریک الکتریکی فراجمجمه‌ای با نویز تصادفی روی قشر پیش پیشانی خلفی جانبی بر بدتنظیمی هیجانی و سرعت پردازش اطلاعات" در مورد اضافه شدن توضیحات مربوط به گروه شم در بخش مداخلات انجام گردید.

با تقدیم احترام
علیرضا محمدی

Save message

General information

Reason for update

فا *empty*

En *empty*

Scientific title

فا بررسی اثربخشی تحریک الکتریکی فراجمجمه‌ای با نویز تصادفی روی قشر پیش پیشانی خلفی جانبی بر بدتنظیمی هیجانی و سرعت پردازش اطلاعات

En Investigating the effectiveness of transcranial random noise stimulation on the dorsolateral prefrontal cortex on emotion dysregulation and information processing speed

Public title

فا بررسی تحریک الکتریکی فراجمجمه‌ای با نویز تصادفی روی قشر پیش پیشانی خلفی

En Investigation of transcranial random noise stimulation on the dorsolateral prefrontal cortex

Acronym

empty

Study design Interventional

Phase N/A

Minimum age 18 year

Maximum age 28 year

Gender Male

Inclusion criteria

فا سن بین 18 تا 28 سال
مذکر باشد
سلامت جسمی
سلامت روانی

En Age between 18 - 28
Be male
Physical health
Mental health

Exclusion criteria

فا اختلال روانی
بیمارانی که در هنگام انتخاب تحت روان درماني بوده اند

En Mental disorder
Patients undergoing psychotherapy at the time of selec

Randomization (investigator's opinion) Randomized

Randomization description

فا برای تصادفی سازی از روش تصادفی سازی محدود از نوع تصادفی سازی بلوکی (بلوک های تصادفی چهارتایی) استفاده خواهد شد و برای این منظور از سیستم آنلاین sealedenvelop.com استفاده می شود که چهار گروه اصلی مداخله را بصورت تصادفی (D و C و B و E و ...) مشخص می کند و سپس فرد اول وارد گروه D، بیمار دوم گروه C و ... تا گروه ها تکمیل شوند.

En For randomization, the restricted randomization method of the type of permuted block randomization (Random quadruple blocks) will be used and for this purpose, the online system sealedenvelop.com will be used, which randomly divides the four main intervention groups (D, C, B, E, ...). Then the first person enters group D, the second patient in group C, etc. until the groups are completed.

Blinding (investigator's opinion)
Single blinded

Blinding description
فا بعد از مشخص شدن گروه های مربوطه، بجز محقق اصلی، هیچ کدام از افراد تحت مطالعه و تمام کساني که با آن ها در تماس هستند از تخصیص گروه های مطالعه اطلاع ندارند. به این منظور، اولا کلیه افراد این تصور را دارند که هر یک مداخله ای را دریافت می کنند در عین حال از نوع مداخله نیز بی اطلاع هستند. ثانیا، سایر همکاران که در طول مسیر مطالعه با بیماران در تماس هستند، از گروه مورد مطالعه و نوع آن اطلاعی ندارند. شرکت‌کنندگان و ارزیاب‌ها نسبت به نوع تحریک (واقعی یا sham) کور خواهند بود. تنظیمات دستگاه به‌گونه‌ای است که ظاهر و حس اولیه تحریک در دو گروه یکسان باشد. در متن رضایت‌نامه صرفاً به وجود دو نوع مداخله اشاره شده و هیچ‌گونه اطلاعاتی که به تشخیص گروه اختصاصی (واقعی یا sham) منجر شود ارائه نشده است. تحریک واقعی و sham هر دو با دستگاه یکسان و به‌صورت برنامه‌ریزی‌شده توسط اپراتوری مستقل اعمال می‌شود.

En After the relevant groups are identified, none of the subjects and all those who come into contact with them, except the principal investigator, are aware of the study group assignment. To this end, first, all subjects are under the impression that each is receiving an intervention, but they are also unaware of the type of intervention. Second, other colleagues who come into contact with the patients throughout the study are unaware of the study group and its type. Participants and assessor[illegible] the type of stimulation (real or sham). The device settin[illegible] initial appearance and sensation of the stimulation are the same in the two groups. The consent form merely mentions the existence of two types of intervention and does not provide any information that would lead to the identification of the specific group (real or sham). Both real and sham stimulation are applied with the same device and in a programmed manner by an independent operator.

Placebo
Used

Assignment
Factorial

Purpose
Supportive

Other design features
فا *empty*
En *empty*

Sample size

Target sample size
Target sample size: **40**
More than 1 sample in each individual: **No**
Number of samples in each individual: ***empty***
Description of samples in each individual - Persian: فا ***empty***
Description of samples in each individual - English: En ***empty***

Actual sample size reached
Actual sample size reached: ***empty***
More than 1 sample in each individual: **No**
Actual sample size in each individual: ***empty***
Description of samples in each individual - Persian: فا ***empty***
Description of samples in each individual - English: En ***empty***

Expected recruitment start date
2025-07-31, 1404/05/09

Expected recruitment end date
2025-12-10, 1404/09/19

Actual recruitment start date
empty

Actual recruitment end date
empty

Trial completion date
empty

Secondary Ids

empty

Ethics committees

Ethics committee

Name of ethics committee
Ethics Committee of Baqiyatallah University of Medical Sciences
کمیته اخلاق دانشگاه علوم پزشکی بقیه الله

Street address
Tehran, Molla Sadra Street, Baqiyatallah University of Medical Sciences
تهران، خیابان ملاصدرا، دانشگاه علوم پزشکی بقیه الله

City
Tehran
تهران

Province
Tehran

Postal code
۱۴۳۵۹۱۶۴۷۱

Approval date 2025-05-25, 1404/03/04

Ethics committee reference number IR.BMSU.BAQ.REC.1404.063

Health conditions studied

Description of health condition studied

فا اختلال شخصیت مرزی

En Borderline Personality Disorder

ICD-10 code F60.3

ICD-10 code description Borderline personality disorder

Primary outcomes

Description

فا نمره شاخص شدت اختلال شخصیت مرزی

En The score of Borderline Personality Disorder Severity Index (BPDSI)

Timepoint

فا قبل از مداخله (پیش آزمون) ، بعد از مداخله (پس آزمون) و یک ماه بعد از مداخله (پیگیری)

En Before the intervention (Pre-test), after the intervention (Post-test) and one month after the intervention (Following)

Method of measurement

فا شاخص شدت اختلال شخصیت مرزی (BPDSI)

En Borderline Personality Disorder Severity Index (BPDSI)

Secondary outcomes

Description

فا مقیاس دشواری های تنظیم هیجانی

En Difficulties in Emotion Regulation Scale

Timepoint

فا قبل از مداخله (پیش آزمون) ، بعد از مداخله (پس آزمون)

En Before the intervention (Pre-test), after the intervention (Post-test)

Method of measurement

فا پرسشنامه مقیاس دشواری های تنظیم هیجانی

En Difficulties in Emotion Regulation Scale Questionnaire

Description
فا آزمون جمع‌بندی متوالی شنیداری با زمان‌بندی مشخص
En PASAT test

Timepoint
فا قبل از مداخله (پیش آزمون) ، بعد از مداخله (پس آزمون)
En Before the intervention (Pre-test), after the intervention (Post-test)

Method of measurement
فا نرم افزار پاسات
En PASAT software

Description
فا تست گارنفسکی
En CERQ test

Timepoint
فا قبل از مداخله (پیش آزمون) ، بعد از مداخله (پس آزمون)
En Before the intervention (Pre-test), after the intervention (Post-test)

Method of measurement
ختی هیجان گارنفسکی
En Cognitive Emotion Regulation Questionnaire

Description
فا تست هیجان گراس
En ERQ test

Timepoint
فا قبل از مداخله (پیش آزمون) ، بعد از مداخله (پس آزمون)
En Before the intervention (Pre-test), after the intervention (Post-test)

Method of measurement
فا پرسشنامه راهبردهای تنظیم هیجان گراس
En Gross Emotion Regulation Strategies Questionnaire

Intervention groups

Description

فا گروه شم: گروه شم شامل افراد سالم: افراد سالمی که هیچگونه مداخله ای دریافت نمی کنند. صرفا دستگاه روی سر آنها قرار گرفته لیکن هیچ گونه تحریکی اعمال نمی گردد.

En Sham group: The sham group includes healthy individuals: healthy individuals who do not receive any intervention. The device is simply placed on their heads but no stimulation is applied.

Category placebo

Description

فا گروه مداخله تحریک: تحریک الکتریکی فراجمجه ای با نویز تصادفی: این یک نوع تحریک غیرتهاجمی مغز است که در آن از برای ایجاد جریان الکتریکی در یک منطقه خاص از مغز از نویز استفاده می شود. در گروه tRNS، تحریک الکتریکی فراجمجه ای با نویز تصادفی با دستگاه شرکت Magstim زیر نظر روانپزشک و فیزیولوژیست مرکز روانپزشکی واقع در بیمارستان امام حسین(ع) انجام خواهد شد. همانطور که اشاره شد، براساس مطالعات پیشین، ناحیه مورد نظر برای این منظور قشـر پیش پیشـانی خلفـی - جانبـی نیمکره راست (DLPFC چپ) با فرکانس 10 هرتز به مدت 20 برای هر فرد حاضر در گروه انجام خواهد شد. بعد از اتمام این مداخله پرسشنامه ها و آزمون پاسات بلافاصله بعد از آن اخذ خواهد شد. و برای حالت تحریک فعال، [illegible] به مدت 20 دقیقه انجام می پذیرد. الکترودهای تحریک به صورت فوق و بر روی ناحیه [illegible] سمت چپ تنظیم می شوند: الکترود مرکزی AF4 و 4 الکترود به صورت دایره ای در نواحی Fz, F4, F8, Fp2 قرار می گیرند.

En Stimulation intervention group: Transcranial electrical stimulation with random noise: This is a type of non-invasive brain stimulation in which noise is used to create an electrical current in a specific area of the brain. In the tRNS group, transcranial electrical stimulation with random noise will be performed with a Magstim device under the supervision of a psychiatrist and physiologist at the Psychiatric Center located at Imam Hussein Hospital (AS). As mentioned, based on previous studies, the area of interest for this purpose is the right dorsolateral prefrontal cortex (left DLPFC) with a frequency of 10 Hz for 20 minutes for each person in the group. After completing this intervention, questionnaires and the PASS test will be taken immediately afterwards. And for the active stimulation mode, stimulation is performed at an intensity of 2mA for 20 minutes. The stimulation electrodes are set as above and on the left DLPFC area: the central

electrode AF4 and 4 electrodes are placed in a circle in the areas Fz, F4, F8, Fp2.

Category treatment-other

Description

فا گروه مداخله برای القای اختلال شخصیت مرزی: اختلال بدتنظیمی هیجانی اطلاعات حال روانی ای است که کمتر تشخیص داده شده و اکثر بیمارانی که به آن مبتلا هستند، شرایط روانی دیگری را نیز به همراه دارن. در این مداخله افراد حاضر در این گروه با مشاهده 50 تصویری که از لحاظ روانی افراد را به هم می ریزد را مشاهده میکنند و با این کار این بدتنظیمی هیجانی بصورت القایی در آنان ایجاد میگردد.

En Intervention group for induction of EUPD: Intervention group: Emotional dysregulation disorder is an underdiagnosed mental health condition, and most patients with it also have other mental health conditions. In this intervention, people in this group view 50 images that are psychologically upsetting, and this emotional dysregulation is induced in them.

Category other

Description

گروه مداخله: بدتنظیمی هیجانی (اختلال شخصیت مرزی) + تحریک الکتریکی فراجمجمه ای با نویز تصادفی. در این گروه بعد از القای بدتنظیمی هیجانی تحریک الکتریکی فراجمجمه ای با نویز تصادفی بروی ناحیه ی DLPFC اعمال خواهد شد.

En Intervention group: Emotional dysregulation (EUPD) + transcranial electrical stimulation with random noise. In this group, after inducing emotional dysregulation, transcranial electrical stimulation with random noise will be applied to the DLPFC region.

Category treatment-other

Recruitment centers

Recruitment center

Name of recruitment center
Psychiatric Hospital 505
بیمارستان روانپزشکی 505

Full name of responsible person
Alireza Mohammadi
دکتر علیرضا محمدی

Street address
Arteh Highway - End of Shahid Mozhdi Boulevard (Oshan) - 505 Hospital
اتوبان ارتش - انتهای بلوار شهید مژدی(اوشان) - بیمارستان ۵۰۵

City
Tehran
تهران

Province
Tehran

Postal code
1411718541

Phone
+98 21 2248 1806

Email
ar.mohammadi@bmsu.ac.ir

Sponsors / Funding sources

Sponsor

Name of organization / entity
Bagheiat-allah University of Medical Sciences

دانشگاه علوم پزشکی بقیه الله

Full name of responsible person
Dr Ali Shiri (Vice President for Research in Bagheiat-allah University of Medical Sciences)
دکتر علی شیری (معاونت پژوهشی دانشگاه بقیه الله)

Street address
C5 uinit, No 5, Kharami Street, Mulla Sadra Street, Vanak Square
میدان ونک، خیابان ملاصدرا، خیابان خوارزمی پلاک 5، واحد C5

City
Tehran
تهران

Province
Tehran

Postal code
1435913111

Phone
+98 21 8862 3188

Email
ar.mohammadi@bmsu.ac.ir

Grant name
فا *empty*
En *empty*

Grant code / Reference number
empty

Title of funding source
فا دانشگاه بقیه الله
En Baqiyatullah University

Proportion provided by this source
1%

Public or private sector
private

domestic

Domestic or foreign origin

Person responsible for general inquiries

Name of organization / entity
Bagheiat-allah University of Medical Sciences
دانشگاه علوم پزشکی بقیه الله

Full name of responsible person
Alireza Mohammadi
دکتر علیرضا محمدی

Position
Associate Professor

Latest degree
Ph.D.

Other areas of specialty/work
Neuroscience
علوم اعصاب

Street address
Vanak Square, Mulla Sadra Street, Sheikh Baha'i Intersection
میدان ونک، خیابان ملاصدرا، تقاطع شیخ بهایی

City
Tehran
تهران

Province
Tehran

Postal code

1435916471

Phone
+98 21 8862 3188

Mobile
+98 912 542 3473

Email
ar.mohammadi@bmsu.ac.ir

Person responsible for scientific inquiries

Name of organization / entity
Bagheiat-allah University of Medical Sciences
ی بقیه الله

Full name of responsible person
Alireza Mohammadi
دکتر علیرضا محمدی

Position
Associate Professor
دانشیار

Latest degree
Ph.D.

Other areas of specialty/work
Neuroscience
علوم اعصاب

Street address
C5 uinit, No 5, Kharami Street, Mulla Sadra Street, Vanak Square
میدان ونک، خیابان ملاصدرا، خیابان خوارزمی پلاک 5، واحد C5

City
Tehran

تهران

Province
Tehran

Postal code
1435916471

Phone
+98 21 8862 3188

Mobile
+98 912 542 3473

Email
ar.mohammadi@bmsu.ac.ir

Person responsible for updating data

Name of organization / entity
Bagheiat-allah University of Medical Sciences

دانشگاه علوم پزشکی بقیه الله

Full name of responsible person
Alireza Mohammadi

دکتر علیرضا محمدی

Position
Associate Professor

دانشیار

Latest degree
Ph.D.

Other areas of specialty/work
Neuroscience

علوم اعصاب

Street address
C5 uinit, No 5, Kharami Street, Mulla Sadra Street, Vanak Square

میدان ونک، خیابان ملاصدرا، خیابان خوارزمی پلاک 5، واحد C5

City
Tehran

تهران

Province
Tehran

Postal code
1435916471

Phone
+98 21 8862 3188

Mobile
+98 912 542 3473

Email
ar.mohammadi@bmsu.ac.ir

Protocol summary

Study aim

فا بررسی اثربخشی تحریک الکتریکی فراجمجمه‌ای با نویز تصادفی روی قشر پیش پیشانی خلفی جانبی بر بدتنظیمی هیجانی و سرعت پردازش اطلاعات

En Investigating the effectiveness of transcranial random noise stimulation on the dorsolateral prefrontal cortex on emotion dysregulation and information processing speed

Participants/Inclusion and exclusion criteria

فا 1. سن افراد بین 18-28 سال 2. راست دست 3. سلامت جسمی و عدم وجود بیماریهای سیستماتیک 4. عدم مصرف منظم هر گونه دارو 5. عدم مصرف سیگار و امثال آن

En 1. Age between 18-28 years 2. Right-handed 3. Physical health and absence of systemic diseasesneurological or mental diseases or disorders 4. No regular use of any medication 5. No smoking or the like

Intervention groups

فا Sham با تحریک پلاسبو، Sham با تحریک tRNS، بدتنظیمی هیجانی القا شده بدتنظیمی القا شده بهمراه تحریک tRNS

En Sham with placebo stimulation, Sham with tRNS stimulation, Emotional dysregulation Emotional dysregulation with tRNS stimulation

Design

فا آزمودنی ها به سوالات پرسشنامه هایی که در قسمت بیان مساله بیان شد، پاسخ می دهند. سپس به چهار گروه تقسیم بندی شدند.در گروه هایی که تحریک انجام میشود، الکترودهای تحریک به صورت فوق و بر روی ناحیه DLPFC سمت چپ تنظیم می شوند: الکترود مرکزی AF4 و 4 الکترود به صورت دایره ای در نواحی Fz, F4, F8, Fp2 قرار می گیرند. مرحله بعدی به عملیات ثبت تسک PASAT انجام خواهد گرفت. برای تحلیل کارکردهای اجرایی و سرعت پردازش اطلاعات در افراد از تست PASAT استفاده خواهد شد و برای تحلیل میزان تنظیم و بدتنظیمی هیجان از پرسشنامه های های (گارنفسکی، ERQ و DERS) کمک گرفته خواهد شد.

En The subjects answered the questionnaire questions stated in the problem statement. Then they were divided into four gr[illegible] where stimulation is performed, the stimulation electrodes are set above and on the left DLPFC area: the central electrode AF4 and 4 electrodes are placed in a circle in the areas Fz, F4, F8, Fp2. The next step will be the PASAT task recording operation. The PASAT test will be used to analyze the executive functions and information processing speed in individuals, and the Hay questionnaires (Garnofsky, ERQ and DERS) will be used to analyze the level of emotion regulation and dysregulation.

Settings and conduct

فا دانشجویان دکتری تخصصی ساکن در خوابگاه دانشگاه علوم پزشکی تهران بعنوان افراد مورد نظر برای طراحی مطالعه انتخاب شده اند. برای تصادفی سازی از روش تصادفی سازی محدود از نوع تصادفی سازی بلوکی (بلوک های تصادفی چهارتایی) استفاده خواهد شد. در مطالعه ی ما چهار گروه پیش بینی شده است که افراد مورد نظر بدون ترتیب و جهت گیری خاصی در چهار گروه تقسیم شده اند. شم، tRNS، اختلال هیجان القایی، اختلال هیجان القایی بهمراه tRNS.

En PhD students residing in the dormitory of Tehran University of Medical Sciences were selected as the subjects for the study design. For

randomization, a limited randomization method of block randomization (quadrant blocks) will be used. In our study, four groups have been predicted, and the subjects have been divided into four groups without any specific order or orientation: Sham, tRNS, induced emotion dysregulation, and induced emotion dysregulation with tRNS.

Main outcome variables

فا تست PASAT تست گازنفسکی تست DERS تست ERQ

En PASAT Test Gasnefsky Test DERS Test ERQ Test

Sharing plan

Deidentified Individual Participant Data Set (IPD)
Yes - There is a plan to make this available

Justification/reason for indecision/not sharing IPD

En *empty*

Study Protocol
Yes - There is a plan to make this available

Statistical Analysis Plan
Yes - There is a plan to make this available

Informed Consent Form
Yes - There is a plan to make this available

Clinical Study Report
Yes - There is a plan to make this available

Analytic Code
Yes - There is a plan to make this available

Data Dictionary
Yes - There is a plan to make this available

Title and more details about the data/document

فا کلیه داده های فردی شرکت کنندگان در مطالعه پس از غیر قابل شناسایی کردن افراد قابل اشتراک گذاری است.

En All personal data of the study participants can be shared after encoding individuals.

When the data will become available and for how long
فا زمان انتشار مستندات داده پس از چاپ مقالات مربوطه در دسترس خواهد.
En Data release time will be available after the relevant articles are published.

To whom data/document is available
فا همه محققین
En All researchers

Under which criteria data/document could be used
فا در صورتی که به مقالات این طرح ارجاع داده شود
En If the articles of this project are cited to.

From where data/document is obtainable
فا علیرضا محمدی
En Alireza Mohammadi

What processes are involved for a request to access data/document
فا با ارسال یک ایمیل به مسئول طرح
En By sending an email to the project manager

Comments
فا *empty*
En *empty*

Trial results

Summary result posting date *empty*

Table of baseline comparison - English

Table of baseline comparison - Persian

Participant flow diagram - English

Participant flow diagram - Persian

Table of variable outcomes' results - English

Table of variable outcomes' results - Persian

Table of adverse events - English

Table of adverse events - Persian

First publication date *empty*

Abstract of published paper
فا *empty*
En *empty*

Link to full English paper *empty*

Link to full Persian paper *empty*

- Home (/)
- About IRCT (/)
- Contact us (/)
- Help (/)

Tel:
Working hours:
8:00 - 15:30 Tehran time
11:30 - 19:00 GMT

0098 21 8670 5503

During COVID-19 Epidemic at working times:
0098 936 770 7834

Fax:
0098 21 8670 5503

Email:
admin@irct.ir (mailto:admin@irct.ir)

Directly contacting the manager:
0098 912 778 2686

Address:
IRCT administration team,
Central Library Building, Iran University Campus,
Hemmat freeway, next to Milad tower,
Tehran, 14496-14535
Iran

Copyright © IRCT 2008-2026. All Rights Reserved.
